# Organ-Specific Quantitative Genetics and Candidate Genes of Phenylpropanoid Metabolism in *Brassica oleracea*

**DOI:** 10.3389/fpls.2015.01240

**Published:** 2016-01-28

**Authors:** Marta Francisco, Mahmoud Ali, Federico Ferreres, Diego A. Moreno, Pablo Velasco, Pilar Soengas

**Affiliations:** ^1^Group of Genetics, Breeding and Biochemistry of Brassicas, Misión Biológica de Galicia - Consejo Superior de Investigaciones Científicas (MBG-CSIC)Pontevedra, Spain; ^2^Department of Horticulture, Faculty of Agriculture, Ain Shams UniversityCairo, Egypt; ^3^Research Group on Quality, Safety and Bioactivity of Plant Foods, Department of Food Science and Technology, Centro de Edafología y Biología Aplicada del Segura - Consejo Superior de Investigaciones Científicas (CEBAS-CSIC)Murcia, Spain

**Keywords:** *Brassica oleracea*, phenylpropanoid metabolism, QTL mapping, candidate gene, flavonoid, hydroxycinnamic acid

## Abstract

Phenolic compounds are proving to be increasingly important for human health and in crop development, defense and adaptation. In spite of the economical importance of *Brassica* crops in agriculture, the mechanisms involved in the biosynthesis of phenolic compounds presents in these species remain unknown. The genetic and metabolic basis of phenolics accumulation was dissected through analysis of total phenolics concentration and its individual components in leaves, flower buds, and seeds of a double haploid (DH) mapping population of *Brassica oleracea*. The quantitative trait loci (QTL) that had an effect on phenolics concentration in each organ were integrated, resulting in 33 consensus QTLs controlling phenolics traits. Most of the studied compounds had organ-specific genomic regulation. Moreover, this information allowed us to propose candidate genes and to predict the function of genes underlying the QTL. A number of previously unknown potential regulatory regions involved in phenylpropanoid metabolism were identified and this study illustrates how plant ontogeny can affect a biochemical pathway.

## Introduction

**Figure 1:**
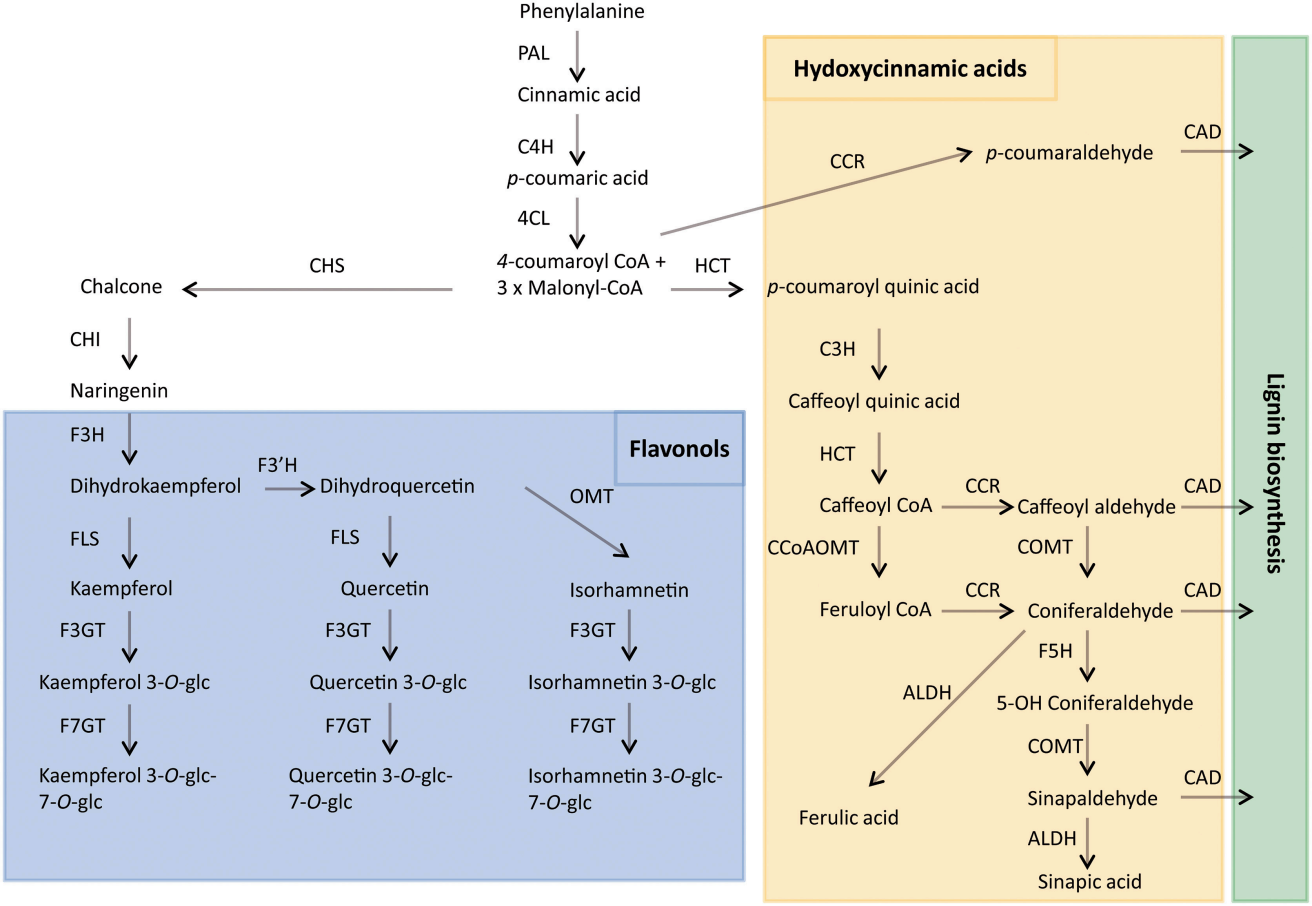
**Proposed phenylpropanoid pathway in the *Brassicaceae* family**. ALDH, aldehyde dehydrogenase; CAD, cinnamyl alcohol dehydrogenase; CCR, cinnamyl CoA reductase; CCoAMT, caffeoyl CoA *O*-methyltransferase; C4H, cinnamate-4-hydroxylase; CHS, chalcone synthase; CHI, chalcone isomerase; COMT, caffeic acid *O*-methyltransferase; C3H, coumarate 3-hydroxylase; 4CL, 4-coumaroyl CoA-ligase; F3GT, flavonol 3-O-glucosyltransferase; F7GT, flavonol 7-*O*-glucosyltransferase; FLS, flavonol synthase; F3H, flavanone 3-hydroxylase; F3'H, flavonoid-3′ hydroxylase; F5H, ferulate 5-hydroxylase; HCT, hydroxycinnamoyl-CoA:shikimate hydroxycinnamoyl transferase; OMT, *O*-methyltransferase; PAL, phenylalanine ammonia-lyase; glc, glucose.

A wide range of secondary metabolites that are synthesized by plants are not required in the primary processes of growth and development but are of vital importance for plant interaction with the environment, for their defense mechanism and for their reproductive strategy (Cheynier et al., 2013). Phenolic compounds are the most widely distributed secondary metabolites, ubiquitously present in the plant kingdom. They are synthesized from phenylalaline via the shikimate/phenylpropanoid pathway (Figure 1). Research in *Arabidopsis* has shown that the pathway starts with the conversion of phenylalanine into cinnamic acid by phenylalanine ammonia lyase (PAL). The cinnamate is hydroxylated by cinnamate 4-hydroxylase (C4H) to form *p*-coumaric acid. The 4-coumarate:CoA ligase enzyme (4CL) then converts *p*-coumaric acid into *p*-coumaroyl CoA by addiction of a CoA thioester, which is the precursor of variuos phenylpropanoid derivatives, including flavonoids, lignins, and isoflavonoids (Fraser and Chapple, 2011). More than 8000 phenolic compounds have been isolated and reported from plant sources which have diverse structural configurations and polarities (Robbins, 2003).

Phenolic compounds in plants are involved in a variety of functions. They can modulate essential physiological processes, such as transcriptional regulation but also they are involved in processes such us growth, development, adaptation, symbiosis, diseases, and responses to pathogen attack (Bhattacharya et al., 2010; Mandal et al., 2010). In humans, phenolic compounds have received considerable attention for being potentially protective factors against cancer and heart diseases (Crozier et al., 2009), in part because of their potent antioxidative properties and their ubiquity in a wide range of commonly consumed foods of plant origin.

The *Brassicaceae* family includes horticultural species consumed by people all over the world considered important food crops in China, Japan, India, and European countries. The main vegetable species of this family is *Brassica oleracea*, which includes many well-known crops such as kale, cabbage, broccoli, Brussels sprouts, and cauliflower. These vegetables are known for their characteristic and high content of secondary metabolites such as glucosinolates (GLS) and phenolic compounds widely studied for its beneficial properties (Cartea et al., 2010; Dinkova-Kostova and Kostov, 2012). For a better understanding of metabolic pathways and their regulations it is important to mention the significance of interactions and possible competition for precursors between GLS pathway and the phenylpropanoid pathways, with respect to environmental effects and genetics (Francisco et al., 2012; Kim et al., 2015).

The most common phenolic compounds presents in *Brassica* are flavonoids derivatives (flavonols and anothocyannins) and hydroxycinnamic acids. The flavonoids consist of 15 carbons with two aromatic rings connected by a three-carbon bridge (C6-C3-C6 carbon skeleton). Among the flavonoids, flavonols of quercetin, kaempferol, and isorhamnetin glycosilated and/or acylated by different hydroxycinnamic acids are the most common in *Brassica* crops. Hydroxycinnamic acids, on the other hand, are aromatic compounds with a three-carbon side chain (C6–C3). Caffeic, ferulic, *p*-coumaric, and sinapic acids are appearing in high quantities in *Brassica*, often found in conjugation with sugar moieties or other hydroxycinnamics (Cartea et al., 2010).

In a green part of *Brassica* vegetables it can be found up to 40 structurally different flavonoids and hydroxycinnamic acids. Moreover, the phenolic profile and concentration may vary between different tissues of the plant and within different populations of the same plant species (Ferreres et al., 2006; Ayaz et al., 2008; Sousa et al., 2008; Francisco et al., 2009). Several studies have found that the regulation of phenol production occurs principally through changes in the transcription rate of the biosynthetic genes (Takos et al., 2006; Pereira et al., 2009; Carbonell-Bejerano et al., 2014; Koyama et al., 2014). During the last years, there is an increasing interest in phenolics regulation. Extensive studies on model organisms like tobacco, *Arabidopsis thaliana*, and *Medicago truncatula* facilitate the understanding of regulation system of the phenolic pathway (Tamagnone et al., 1998; Pang et al., 2008; Zhao and Dixon, 2009; Verdier et al., 2012). However, in other plant species, the genetic control of phenolics production has been the subject of very few genetic studies. In *Brassica* vegetables the majority of them are focused in the molecular regulation of sinapine metabolism in oilseed rape (*B. napus* L. var. *napus*) (Milkowski et al., 2004; Baumert et al., 2005). Seeds of oilseed rape accumulate high amounts of sinapate esters. Given the antinutritive properties that these metabolites confer on the protein fraction, low sinapate ester content is one major aim of breeding programs designed to increase the nutritional value of seeds from *B. napus.* Based on the absence of soluble sinapate esters in the *A. thaliana* mutant *sin1*, the homologous gene from *B. napus* was identified and the sequence information was used for antisense suppression strategy to obtain lines with reduced levels of sinapate esters (Nair et al., 2000). Comprehensive analysis of transgenic seeds with other silenced genes involved in sinapate ester biosynthesis revealed that both the amount of sinapoylglucose and that of the minor sinapate esters can be drastically reduced to trace amounts (Hüsken et al., 2005). In a more recent study, Rezaeizad et al. (2011) detected SSR markers linked to phenolic compounds also in oilseed rape using an association mapping approach, which could be employed in a marker assisted selection.

In spite of the economical importance of *Brassica* species in agriculture, the mechanisms involved in the biosynthesis of defense and/or health-related phenolic compounds presents in these plants remain unknown. This study aims to identify genomic regions controlling phenolic composition and content in three different organs (leaves, flower buds, and seeds) in a double haploid (DH) population of *B. oleracea*. We utilized a quantitative trait locus (QTL) mapping approach to better understand the genetic basis of phenolic accumulation within plant ontogeny. For major QTL regions, candidate genes were proposed using two different approaches, by *in silico* search in the *B. oleracea* available sequences and by genome comparison with *A. thaliana* through analysis of syntenic regions.

## Materials and methods

### Plant material

A double haploid (DH) mapping population (BolTBDH) was employed in this work. The population was created from an F_1_individual from a cross between a DH rapid cycling of Chinese kale (TO1000DH3, P_1_) and a DH broccoli line “Early Big” (P_2_) (Iniguez-Luy et al., 2009). Parents and 155 DH inbred lines were sown in a greenhouse under 16 h of daylight and a temperature of 24 ± 2°C, and 8 h of darkness with 18 ± 2°C at night, and a relative humidity of 55%. Plants were sown in a completely randomized experiment with two replications and four plants per replication and DH line. From each line, leaf samples were taken at the 4 leaves stage and flower buds were taken differentially depending on the flowering time of each plant. One bulk was taken from each replication by mixing the four samples of leaves and flower buds. Samples were immediately conserved at −80°C, and afterwards they were lyophilized for 48 h (Christ® Beta 2-8 LD Plus Freeze Dryer, Germany). The dried material was powdered using an IKA-A10 (IKA-Werke GmbH & Co.KG) mill and the powder was used for analysis. Besides, two bulks of 50 mg of seed for each line were prepared for phenolic analysis.

### Phenolic extraction

Fifty milligrams of each sample were extracted in 500 μL 70% methanol and sonicated during 1 h (model: 3510E-MTH, Bransonic®, Mexico) to facilitate the extraction. The suspensions were allowed to stand overnight at 4°C and afterwards they were sonicated again for 1 h. Then, samples were centrifugated at 3700 rpm for 15 min. Supernatants were recovered and were filtered through 0.22 μm PTFE filters (Multi Screen®, Ireland).

### Identification of phenolic compounds by HPLC-DAD-ESI/MS^n^ analysis

For phenolics identification, three samples from each parent at each plant organ were analyzed. Besides, we identified these compounds in samples from one inbreed line from the mapping population (TODH101). The UV spectra analysis was carried out on a Kinetex column (5 μm, C18, 100 A, 150 × 4.6 mm; Phenomenex, Macclesfield, UK). The mobile phase consisted of two solvents: water-acetic acid (1%) (A) and methanol (B), starting with 20% B and using a gradient to obtain 40% B at 20 min and 60% B at 30 min. The flow rate was 1 mL/min, and the injection volume was 20 μL. Spectral data from all peaks were accumulated in the range of 240–400 nm, and chromatograms were recorded at 330 nm. The HPLC-DAD-ESI/MS^n^ analyses were carried out in an Agilent HPLC 1100 series equipped with a diode array detector and mass detector in series (Agilent Technologies, Waldbronn, Germany). The HPLC consisted of a binary pump (model G1312A), an auto sampler (model G1313A), a degasser (model G1322A), and a photodiode array detector (model G1315B). The HPLC system was controlled by ChemStation software (Agilent, v. 08.03). The mass detector was an ion trap spectrometer (model G2445A) equipped with an electro spray ionization interface and was controlled by LCMSD software (Agilent, v. 4.1). The ionization conditions were adjusted at 350°C and 4 kV for capillary temperature and voltage, respectively. The nebulizer pressure and flow rate of nitrogen were 65.0 psi and 11 L/min, respectively. The full scan mass covered the range from m/z 100 up to *m/z* 1600. Collision-induced fragmentation experiments were performed in the ion trap using helium as the collision gas, with voltage ramping cycles from 0.3 up to 2 V. Mass spectrometry data were acquired in the negative ionization mode. Individual phenolic compounds were identified based on the data obtained from the standard substances or published literature including tR, λmax, ([M–H]^−^), and major fragment ions (Vallejo et al., 2004; Ferreres et al., 2006; Francisco et al., 2009; Velasco et al., 2011).

### Quantification of phenolic compounds by UHPLC

Quantification of phenolic compounds was carried out in the two parents and in the 155 DH inbred lines of the mapping population. An Ultra-High-Performance Liquid-Chromatograph (UHPLC Nexera LC-30AD; Shimadzu) equipped with a Nexera SIL-30AC injector and one SPD-M20A UV/VIS photodiode array detector was used for the quantification. The UHPLC column was a Kinetex™ 2.6 μm C18 82–102 Å, LC Column 100 × 4.6 mm, protected with a C18 guard cartridge. The flow rate was 0.4 mL/min and the oven temperature was set at 30°C. The mobile phase consisted of two solvents: water-acetic acid (1%) (A) and methanol (B), starting with 10% B and using a gradient to obtain 40% B at 15 min and 60% B at 24 min. The injection volume was 5 μL. Chromatograms were recorded at 330 nm and data was processed on a computer with the LabSolutions software (Shimadzu). Caffeoyl quinic and *p*-coumparoyl quinic acids derivatives were quantified as chlorogenic acid (Sigma–Aldrich Chemie GmbH, Steinheim, Germany), flavonoids as kaempferol-3-*O*- glucoside and sinapic acid and derivatives as sinapic acid (Sigma–Aldrich Chemie GmbH, Steinheim, Germany).

### Statistical analysis

A combined analysis of variance across organs and individual analyses of variance for each organ were made for individual and total phenolics. Lines and organs were considered as fixed factors and replications were considered as random factor. Comparisons between parents for each of the studied traits were accomplished with the Student's *t*-test. All the statistical analyses were performed with the PROC GLM of SAS (SAS Institute Inc., 2011).

The genetic map employed for the QTL analysis was created by Iniguez-Luy et al. (2009). It has 279 markers (SSRs and RFLPs) distributed along nine linkage groups assigned to chromosomes C1–C9 of *B. oleracea* with a total distance of 891.4 cM and a marker density of 3.2 cM/marker. Quantitative trait locus mapping was carried out through a composite interval mapping method (Zeng, 1994) by using the software PLABQTL (version 1.2) (Utz, 2003). Individual analyses were carried out for each trait and plant developmental stage (leaf, flower bud and seed). Empirical thresholds were build with permutation tests, with *N* = 1000 (Van Ooijen, 1999). The confidence intervals were set to 95%. The analysis and cofactor election were carried out by following PLABQTL's recommendations. The proportion of phenotypic variance explained for a specific trait was determined by the adjusted coefficient of determination of regression (R^2^) fitting a model which includes all detected QTLs (Papst et al., 2004). Fivefold cross-validation of QTLs was performed by following the procedures described by Utz et al. (2000). The frequency of QTL detection gives us an estimation of the precision of QTL localization. Significant QTLs for phenolic compounds were integrated by using a QTL meta-analysis with BioMercator 4.2 software in order to give consensus QTLs (Goffinet and Gerber, 2000). An Akaike-type statistical criterion (AIC value) indicated the model which best fitted the data, including the number and the consensus QTLs positions. The aim of performing a meta-analysis was to find if a genomic region could determine the content of different phenolic compounds.

### Candidate gene identification

In order to identify candidate genes which may directly account for QTLs in *B. oleracea* we used two different approaches, by *in silico* search in the *B. oleracea* available sequences and by genome comparison with *A. thaliana* through analysis of syntenic regions. DNA sequences codes of the SSRs and RFLPs markers of the BolTBDH mapping population were collected from http://www.brassica.info/resource/markers.php and DNA sequences were obtained from NCBI data base. A BLAST search at the *B. oleracea* Genomics Project web site, Bolbase was done (http://www.ocri-genomics.org/bolbase/index.html) using the DNA sequences of the markers. Two sequences were declared putative homologous if the *E* value of their alignment was = 10^−4^. Then, genes related to phenylpropanoid pathway were searched in those regions corresponding to the confidence interval of each QTL (Table S1). This approach was not possible to use for all the identified QTLs since the *B. oleracea* genome for download is yet incomplete. Then we also perform a comparative mapping study of *B. oleracea* and *A. thaliana*. Iniguez-Luy et al. (2009) identified collinear genomic blocks between the mapping population used in this study (BolTBDH) and *A. thaliana* by using a synteny analysis. Following this approach, we tried to locate genes involved in phenylpropanoid metabolism in *A. thaliana* which were obtained from TAIR (The *Arabidopsis* Information Resource) on the BolTBDH map by *in silico* mapping (Table S2).

## Results

### Metabolite profiling and phenotype variation in the *B. oleracea* BolTBDH mapping population

**Figure 2:**
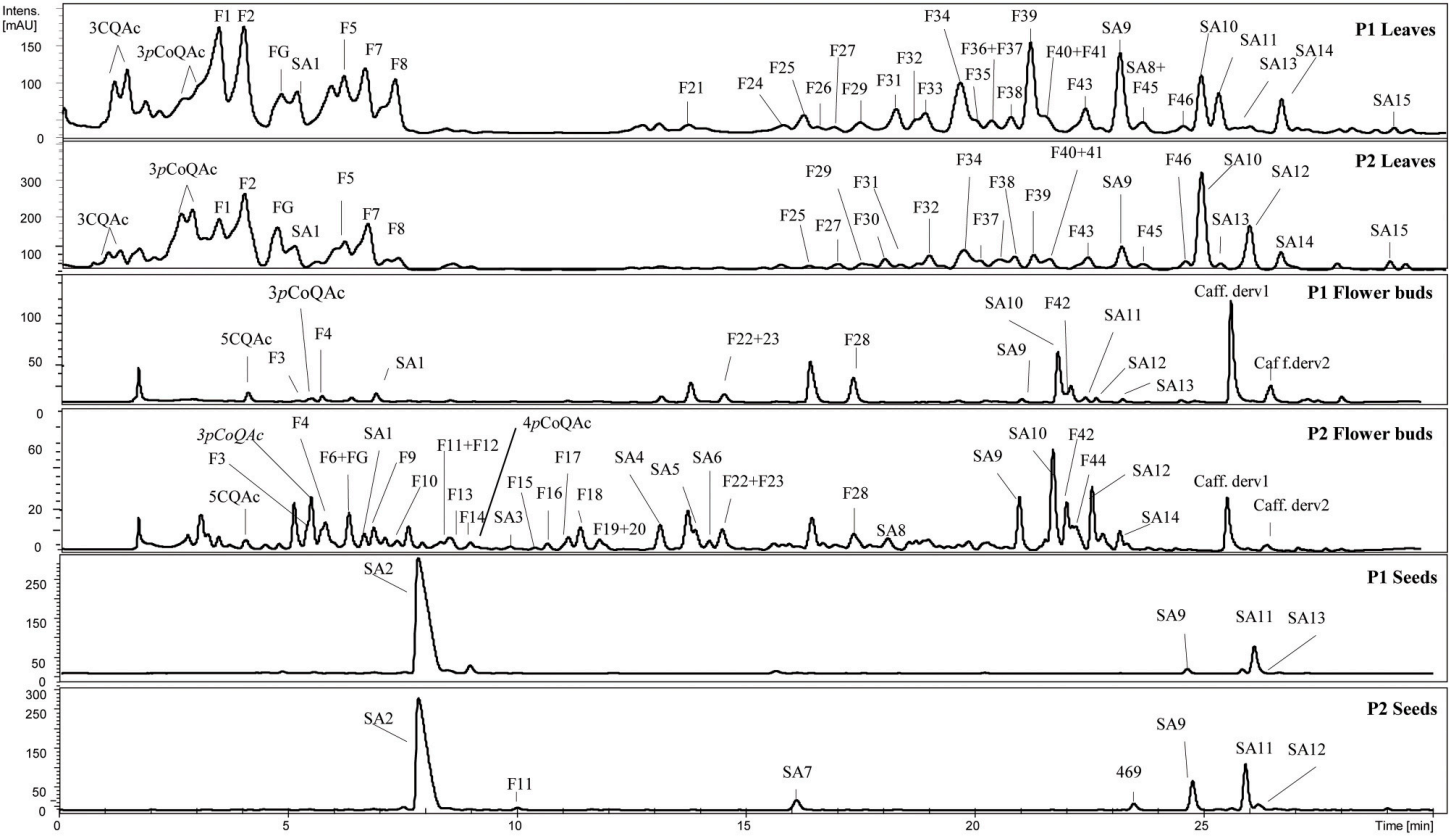
**Phenolic profile in leaves, flower buds and seeds of a double haploid population of *Brassica oleracea*; P_1_ (TO1000DH3) and P_2_ (“Early Big” broccoli)**. See Table S4 for compounds abbreviations.

A total of 36, 38, and 7 phenolic compounds, including flavonoids and hydroxycinnamic acids, were identified in the leaves, flower buds and seeds of the BolTBDH mapping population, respectively (Figure 2). The flavonoids were identified as *O*-glycosides containing a substituent at 3− and/or 7− positions of quercetin, kaempferol, and isorhamnetin and/or in conjugation with different hydroxycinnamic acids. Acylation were most frequently at the 3-position. The identified hydroxycinnamic acids included caffeoyl and *p*-coumaroyl quinic acids, feruloyl glycoside, and different sinapoyl derivatives glycosilated with at least two sugar moieties (gentiobiosides).

The major individual phenolics in each plant organ under study were then quantified in both parents and in the 155 inbred lines of the DH population. Fifteen individual phenolic compounds were quantified in the leaves samples, 15 in the flower buds, and five in the seeds. The mean of each individual phenolic compound quantified in the two parents and in 155 lines from the mapping population at the three plant organs under study are shown in Table 1. Besides individual compounds, total flavonoids, total hydroxycinnamic acids and total phenolics contents were also computed. At each plant stage, the two parents exhibited a similar phenolic profile but differed on their content. In leaves and flower buds, total phenolic content was higher in P_2_ than in P_1_. In contrast, P_1_ showed higher phenolics content than P_2_ in the seeds.

**Table 1:** **Individual and total phenolics content of parents and in the 155 lines of BolTBDH mapping population in three different plant organs**.

	**Leaves**				**Flower buds**				**Seeds**			
**Compound**	**P1^a^**	**P2^b^**	**Mean (range)^c^**	***t*-test^d^**	**P1^a^**	**P2^b^**	**Mean (range)^c^**	***t*-test^d^**	**P1^a^**	**P2^b^**	**Mean (range)^c^**	***t*-test^d^**
**FLAVONOIDS**												
F1	1.02 ± 0.38	3.73 ± 1.74	2.52 (0–9.91)	ns	–	–	–	–	–	–	–	–
F2	0.59 ± 0.02	1.72 ± 0.07	1.15 (0–5.21)	^*^	–	–	–	–	–	–	–	–
F5	0.26 ± 0.14	1.06 ± 0.04	0.84 (0–3.44)	^*^	–	–	–	–	–	–	–	–
F7	0.15 ± 0.08	0.30 ± 0.03	0.34 (0–6.23)	^*^	–	–	–	–	–	–	–	–
F8	–	–	0.18 (0–2.4)	–	–	–	–	–	–	–	–	–
F11	–	–	–	–	–	5.28 ± 0.31	1.01 (0–5.43)	–	1.71 ± 0.12	0.10 ± 0.07	0.37 (0.03–1.79)	^*^
F15	–	–	–	–	0.06 ± 0.04	0.25 ± 0.11	1.79 (0–6.97)	ns	–	–	–	–
F16	–	–	–	–	0.19 ± 0.26	2.12 ± 0.17	2.05 (0–9.59)	^*^	–	–	–	–
F18	–	–	–	–	0.34 ± 0.48	5.09 ± 0.13	3.68 (0.18–15.32)	^*^	–	–	–	–
F23	–	–	–	–	3.17 ± 0.31	9.97 ± 1.87	3.95 (0–13.44)	^*^	–	–	–	–
F25	0.26 ± 0.13	0.12 ± 0.05	1.36 (0–8.16)	ns	–	–	–	–	–	–	–	–
F28	–	–	–	–	3.9 ± 1.83	12.52 ± 1.84	5.58 (0.10–34.19)	^*^	–	–	–	–
F34	0.27 ± 0.06	1.39 ± 0.19	1.35 (0–6.16)	^*^	–	–	–	–	–	–	–	–
F37	0.37 ± 0.17	1.33 ± 0.27	1.54 (0.27–5.81)	^*^	–	–	–	–	–	–	–	–
F44	–	–	–	–	0.34 ± 0.48	10.9 ± 1.93	0.96 (0–4.44)	^*^	–	–	–	–
FlavT	2.92 ± 0.98	9.65 ± 2.40	9.28 (0.33–48.71)	^*^	8.0 ± 1.40	46.13 ± 5.61	19.02 (0.31–83.53)	^*^	1.71 ± 0.12	0.10 ± 0.07	0.37 (0.03–1.79)	^*^
**HYDROXYCINNAMIC ACIDS**												
3CQAc	3.74 ± 0.40	1.31 ± 0.83	4.20 (0.20–20.24)	^*^	–	–	–	–	–	–	–	–
5CQAc	–	–	–	–	0.56 ± 0.52	6.9 ± 1.78	2.45 (0–15.57)	^*^	–	–	–	–
Caff. derv1	–	–	–	–	0.56 ± 0.52	6.9 ± 1.78	2.45 ± (0–15.57)	^*^	–	–	–	–
FG	1.9 ± 0.65	1.39 ± 0.82	1.37 (0.06–5.24)	ns	–	–	–	–	–	–	–	–
SA1	3.63 ± 1.51	1.09 ± 0.21	0.79 (0–2.58)	ns	2.00 ± 0.45	3.63 ± 0.67	3.31 (0.33–24.28)	^*^	–	–	0.67 (0–4.82)	–
SA2	–	–	–	–	–	–	–	–	75.34 ± 2.55	39.96 ± 1.68	67.50 (15.14–187.21)	^*^
SA9	4.42 ± 0.23	6.68 ± 0.96	6.66 (0.14–16.08)	^*^	0.98 ± 0.39	1.52 ± 0.54	5.79 (0–32.25)	ns	1.74 ± 0.45	4.10 ± 0.85	3.18 (0–44.93)	^*^
SA10	3.31 ± 0.04	7.22 ± 0.69	6.86 (0.04–17.42)	^*^	1.04 ± 0.38	12.66 ± 2.81	7.18 (0–14.61)	^*^	–	–	–	–
SA11	0.58 ± 1.10	2.38 ± 0.16	2.17 (0–5.89)	ns	10.14 ± 0.77	10.61 ± 2.20	6.95 (0.53–15.49)	ns	9.69 ± 1.41	12.63 ± 2.51	3.37 (0–38.95)	ns
SA12	–	–	–	–	0.29 ± 0.20	6.22 ± 2.28	3.09 (0–10.71)	^*^	–	–	–	–
HydroxT	18.18 ± 2.69	26.88 ± 0.28	26.33 (0.64–87.68)	^*^	18.14 ± 0.96	63.75 ± 6.45	34.47 (1.14–144.48)	^*^	86.77 ± 3.58	56.69 ± 2.03	74.72 (0.02–307.81)	^*^
PhenolT	21.1 ± 2.76	36.53 ± 4.93	35.61 (0.78–91.88)	^*^	26.14 ± 4.36	109.88 ± 12.05	53.49 (1.45–228.01)	^*^	88.48 ± 3.96	56.79 ± 1.96	75.09 (0.03–309.23)	^*^

a Mean value of the parent P1 (TO1000DH3) ± standard deviation (μmol/g dw).

b Mean value of the parent P2 (“Early Big” broccoli) ± standard deviation (μmol/g dw).

c Mean value and range of the BolTBDH mapping population (μmol/g dw).

d Independent Student's t-test; ^*^statistically significant difference (p < 0.05) between the two parents on the same plant organ. ns, not significant.

See Table S4 for compounds abbreviations.

**Figure 3:**
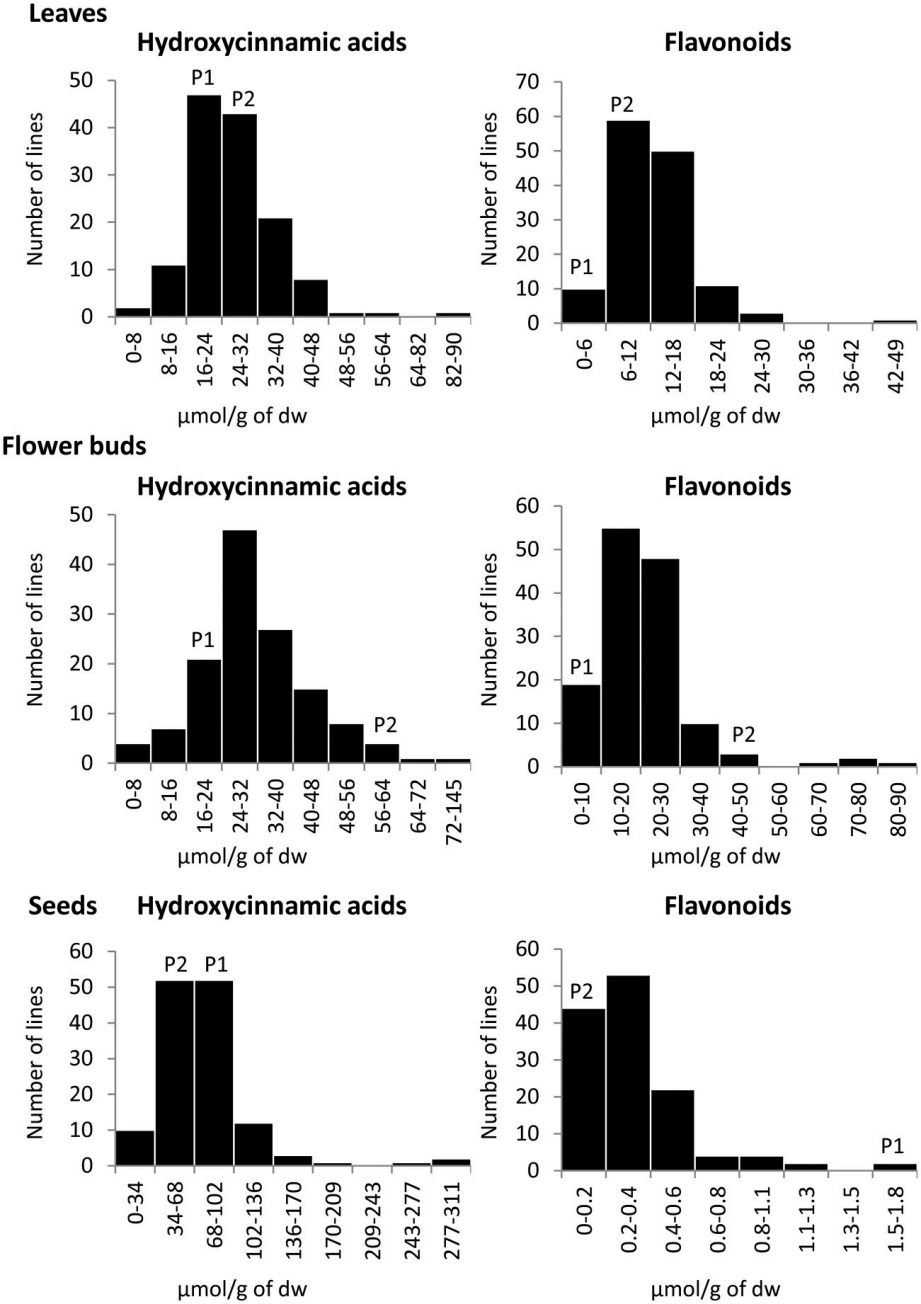
**Frequency distributions of total hydroxycinnamic acids and total flavonoids in the 155 lines of the BolTBDH mapping population and their parental lines in three plant organs (leaves, flower buds, and seeds)**. The vertical axis of each figure represents the number of DH lines. The parental values are the mean of two replicates, indicates as P1 (TO1000DH3) and P2 (“Early Big” broccoli).

The frequency distributions of individual compounds as well as of total flavonoids and total hydroxycinnamic acids content in three plant organs were relatively normal (Figure 3). Transgressive distributions were observed for almost all the individual phenolics quantified in leaves and seeds, meaning that values for the DH mapping population were substantially larger and/or smaller than both of the parents. For instance, the content of the flavonoid F25 in leaves was 0.26 μmol g^−1^ dw in P_1_ and 0.12 μmol g^−1^ dw in P_2_. The average content of this kaempferol derivative in the mapping population was 1.36 μmol g^−1^ dw and ranged from 0 to 8.16 μmol g^−1^ dw (Table 1).

### Phenolic composition differs across plant organs

**Figure 4:**
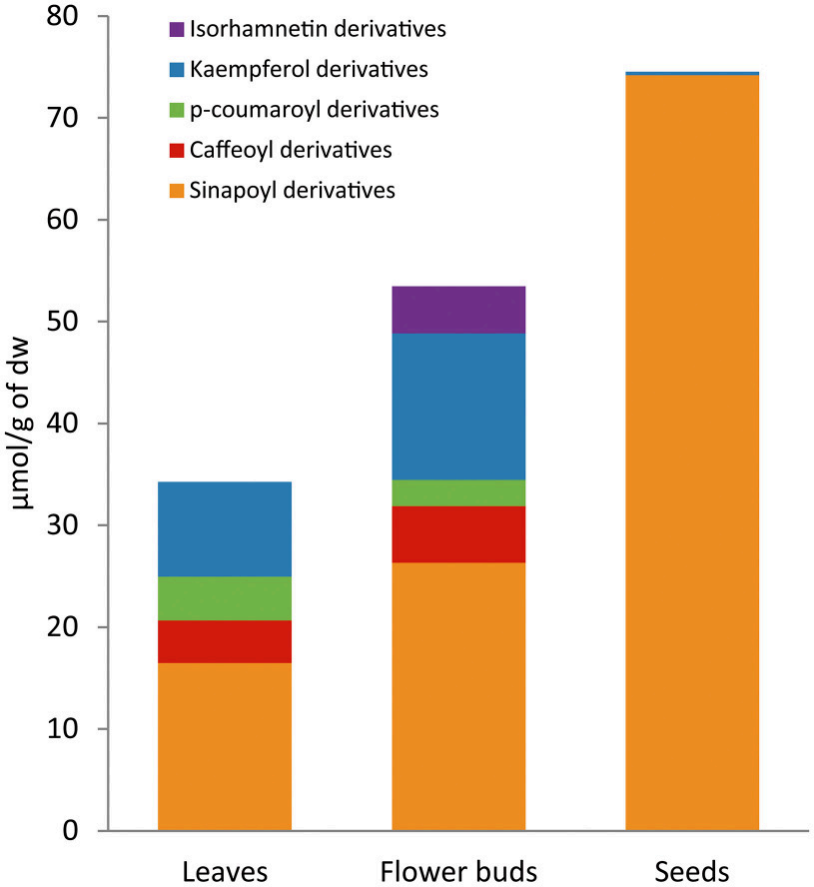
**Phenolic composition differs across plant organs in *Brassica oleracea***. Average content of flavonoids (kaempferol and isorhamnetin derivatives) and hydroxycinnamic acids (sinapoyl, *p*-coumaroyl, and caffeoyl derivatives) in leaves, flower buds, and seeds of the 155 lines from BolTBDH mapping population.

The phenolic profile and content of the mapping population varied depending on the plant organ analyzed (Figure 4). The average content of total phenolics in the population was 35.61 μmol g^−1^ dw in leaves, 53.49 μmol g^−1^ dw in flower buds and 75.09 μmol g^−1^ dw in seeds. In leaves, 26% of total phenolics content was due to flavonoids and 73% to hydroxycinnamic acids. In flower buds, 36% of total phenolics content was due to flavonoids and 64% to hydroxycinnamic acids. On the other hand, seeds showed a completely different phenolic profile with total hydroxycinnamic acids accounting for almost 100% of the total phenolic content.

Across plant organs, flavonoids reached the higher content at flower bud stage (19.02 μmol g^−1^ dw) rather than leaves (9.28 μmol g^−1^ dw). Seeds showed very low quantities of flavonoids, less than 1 μmol g^−1^ dw. The flavonoid profile was also different between plant organs (Figure 2; Table 1). None of the identified flavonoids was coincident between leaves and flower buds. In leaves, all the quantified flavonoids were kaemperol-3-*O*-di/triglucoside-7-*O*-glucoside acylated with one or more hydroxycinnamic acids (F1, F2, F5, F7, F8, F25, F34, and F37). In flower buds, the major flavonoids were kaempferol-3-*O*-diglucoside, kaempferol-3-*O*-glucoside-7-*O*-glucoside and isorhamnetin-3-*O-*diglucoside-7-*O-*glucoside, and in contrast with the flavonoids found in leaves, none of them was in conjugation with hydroxycinnamic acids (F11, F15, F16, F18, F23, F28, and F44). Besides, isorhamnetin derivatives were not present in leaves. Flavonoids of quercetin were identified in both plant organs, but as they appeared in small quantities were not quantified.

Total hydroxycinnamic acids reached the highest average levels in seeds (74.72 μmol g^−1^ dw) and they were mainly represented by only one compound; a putative sinapoyl derivative (SA2) (Table 1). This compound was found exclusively in this plant organ, representing 91% of total phenolic content. The hydroxycinnamic acids profile was similar between leaves and flower buds although differed by content. The sinapoyl gentiobiosides derivatives SA9, SA10, and SA11 were the major phenolic acids in both plant organs. The average content of SA9 was higher in leaves while the content of SA10 and SA11 was higher in flower buds (Table 1).

### Organ-specific QTLs and phenylpropanoid pathway candidate genes

**Figure 5:**
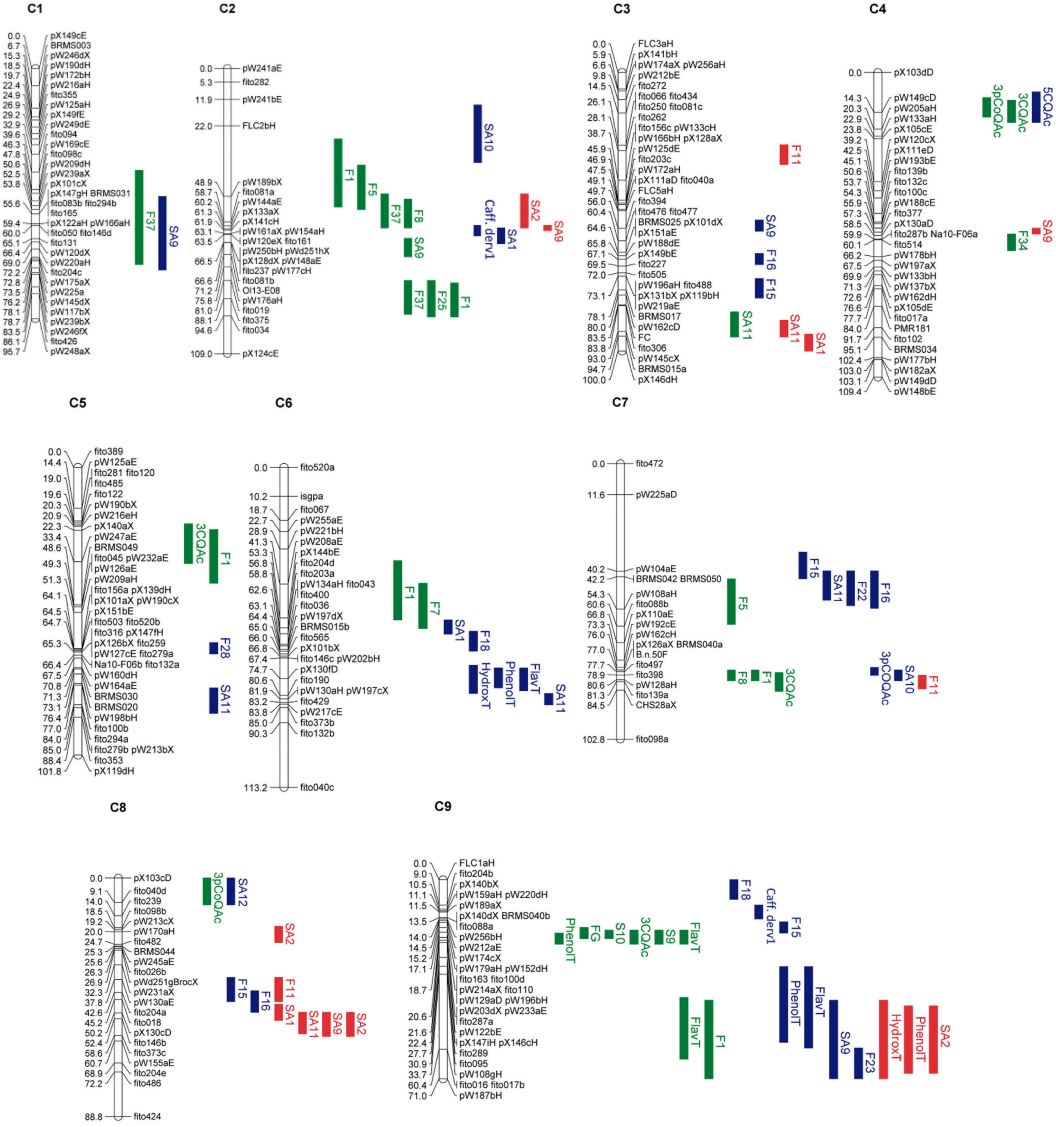
**Linkage map of the BolTBDH mapping population**. Marker locus names are on the right side of each linkage group and distances (cM) are shown for each marker interval. Colors indicate the organ where the QTL was found: green for leaves; blue for flower buds; red for seeds. See Table S4 for compounds abbreviations.

To investigate the genetic control of quantitative variation of phenolics accumulation in leaves, flower buds and seeds of the DH population of *B. oleracea*, a QTL analysis was performed. Detailed information of identified QTLs is given in Table S3 and Figure 5. Seventy nine significant QTLs were detected for the 29 traits analyzed which included the individual phenolics quantified as well as the sum of flavonoids (FlavT), the sum of hydroxycinnamic acids (HydroxT), and total phenolics (PhenolT) in each plant organ. The QTLs were spread all over the 9 linkage groups. These QTLs were not equally distributed over the *B. oleracea* linkage groups, as hotspots for the genetic control of metabolite content could be identified (Figure 5). The number of QTLs by linkage group ranged between two in C1 and 18 in C9. QTLs were detected for all the traits analyzed unless for F2 and F44. In leaves, 30 significant QTLs controlling phenolics content were found. The value of R^2^ ranged between 6.23% for F37 in C1 and 46.05% for 3CQAc in C4. Seventeen QTLs had a frequency of cross-validation higher than 50% (Table S3). In flower buds, 33 QTLs were found. The value of R^2^ ranged between 1.32% for SA11 in C5 and 36.79% for 5CQAc in C4. Fifteen QTLs had a frequency of cross-validation higher than 50% (Table S3). Sixteen QTLs were found in seeds. The value of R^2^ ranged between 3.18% for total hydroxycinnamic acids in C9 and 30.74% for SA1 in C3. Five QTLs had a frequency of cross-validation higher than 50% (Table S3).

The meta-analysis successfully reduced the total QTL by 58%. In total, 33 independent consensus QTL were identified at the three plant organ under study spread all over the nine linkage groups (Table 2). There were six on C3; five on C2 and C9; four on C8; three on C4, C5, C6, and C7; and two on C1.

**Table 2:** **Description of organ-specific consensus quantitative trait loci (QTL) found in BolTBDH mapping population**.

**Linkage group^a^**	**No of consensus QTL**	**Peak position (cM)^b^**	**Confidence interval (cM)^b^**	**F1**	**F2**	**F5**	**F7**	**F8**	**F11**	**F15**	**F16**	**F18**	**F23**	**F25**	**F28**	**F34**	**F37**	**F44**	**FlavT**	**3CQAc**	**5CQAc**	**Caff. derv1**	**3pCoQAc**	**FG**	**SA1**	**SA2**	**SA9**	**SA10**	**SA11**	**SA12**	**HydroxT**	**PhenolT**
1	1.1	42	39–35																													
1	1.2	75	49–77																													
2	2.1	19	14–36																													
2	2.2	54	50–57																													
2	2.3	61	60–63																													
2	2.4	70	67–73																													
2	2.5	87	83–91																													
3	3.1	27	26–33																													
3	3.2	56	53–57																													
3	3.3	67	65–69																													
3	3.4	78	74–81																													
3	3.5	93	86–95																										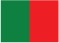			
3	3.6	99	94–100																													
4	4.1	14	7–18																													
4	4.2	60	58–64																													
5	5.1	28	24–32																													
5	5.2	64	62–66																													
5	5.3	82	78–87																													
6	6.1	51	45–58																													
6	6.2	58	56–60																													
6	6.3	78	76–79																													
7	7.1	42	40–45																													
7	7.2	53	43–60																													
7	7.3	79	76–80																													
8	8.1	3	0–10																													
8	8.2	19	18–24																													
8	8.3	41	37–46																													
8	8.4	52	50–54																													
9	9.1	0	0–7																													
9	9.2	14	9–16																													
9	9.3	20	19–21																													
9	9.4	43	35–51																													
9	9.5	60	53–68																													

Colors indicate the organ where the QTL was found: green for leaves; blue for flower buds; red for seeds.

a Linkage group numbers are related to B. oleracea chromosomes 1 to 9.

b cM, centimorgan.

See Table S4 for compounds abbreviations.

Twenty three of the consensus QTLs were plant organ-specific, 13 were found exclusively in flower buds, six in leaves, and four in seeds (Table 2). Some of them were also phenolic kind specific, i.e., QTL-2.5 and QTL-6.1 regulated only flavonoid content in leaves. Moreover, there were found genomic regions were the same QTL regulate the content of more than one compound in a specific organ. In leaves, the consensus QTL-9.3 was found to be responsible for the 8–15% of the phenotypic variation of six phenolic traits. In this region of *B. oleracea* genome a cluster of three putative hydroxycinnamoyl-Coenzyme A shikimate/quinate hydroxycinnamoyltransferases (HCT) was located (Table S1). In flower buds, the consensus QTL-6.3, QTL-7.1, and QTL-9.4 explained 8–12% of the phenotypic variation for different traits including total phenolic content. In the confidence interval of QTL-7.1 two candidate genes codifying for two UDP-glucosyltransferases were found (Table S1). In seeds, the consensus QTL-8.4 was exclusively related with the accumulation of the major hydroxycinnamic acids presents in this plant organ and was responsible for 11–18% of these compounds phenotypic variation.

QTL-7.3 and QTL-9.5, overlapped between the three plant organs. These QTLs were related with up to seven traits, including individual flavonoids and hydroxycinnamic acids as well as total phenolics (Table 2). These QTLs explained 6–30% of the phenolics accumulation among the three plant organs. A putative caffeoyl CoA *O*-methyltransferase was proposed as candidate gene for QTL-7.3 phenotypic variation (Table S1).

Leaves and flower buds showed other three co-located consensus QTLs in common in the linkage groups C4, C7, and C8 (Table 2). The QTL-4.1 was exclusively related with the accumulation of the chlorogenic acids (3CQAc, 5CQAc) and 3-*p*-coumaroyl quinic acid (3*p*CoQAc). This QTL was found to be responsible for a large proportion of phenotypic variation, 36–46%. Alleles for increasing the content of the chlorogenic acids are given by P1, while alleles for increasing the content of the 3*p*CoQAc are given by P2. In the confidence interval of this QTL we found synteny with chromosome 2 of *A. thaliana*. In this region of the model plant was found a key gene of the phenylopropanoid pathway (*AT2g40890*) which codifies a coumarate 3-hydroxylase enzyme (C3H) (Table S2).

Other consensus QTLs co-located between plant organs. The QTL-2.2, QTL-3.5, and QTL-4.2 were coincident for leaves and seeds. The QTL-2.3 and QTL-8.3 were related with the accumulation of different phenolics in both flower buds and seeds (Table 2).

## Discusion

The phenylpropanoid pathway serves as a rich source of metabolites in plants, being required for the biosynthesis of lignin, and serving as a starting point for the production of many other important compounds, such as the flavonoids and hydroxycinnamic acids. These compounds perform a variety of functions in the plant, generally centered on responses to pathogen attach and UV protection, as well as contributing toward the color and sensory characteristics of vegetables (Pereira et al., 2009). Besides, diets rich in foods containing phenolic compounds, such as *B. oleracea* crops, have been reported to possess many useful properties for human health including anti-inflammatory, enzyme inhibition, antimicrobial, antiallergic, vascular and cytotoxic antitumor activity, but the most important action of phenolics is their antioxidant activity (Crozier et al., 2009; Jahangir et al., 2009). In recent years, there is growing interest to increase the content of phytonutrients in crop species. Both traditional plant breeding and biotechnology-based techniques involving modifications of key enzymes catalyzing the synthesis of target compounds are needed to produce plants with the desired quality traits. Therefore, understanding phenolics regulation is one of the great challenges of modern plant biology. In the present work, we identified genomic regions controlling individual and total phenolic compounds accumulation in the *B. oleracea* TO1000DH3 × “Early Big” broccoli DH population. To gain insight into the biological relationship between the metabolism of phenolic compounds and plant development, the accumulation of phenolic compounds were comprehensively studied in three different organs (leaves, flower buds and seeds). To the best of our knowledge, this the first report dissecting genomic regions regulating individual and total phenolic compounds in different plant organs in *B. oleracea*. Moreover, for some of the described QTLs it was possible to propose candidate genes involved in phenylpropanoid metabolism that could control the phenotypic variation of the QTL.

### Metabolite profiling and phenotype variation in the *B. oleracea* BolTBDH mapping population

The main flavonoids identified were kaempferol, isorhamnetin and quercetin derivatives. The chemical structure more common in our samples were flavonol-3-*O*-(cinnamoyl)glycoside-7-*O*-glycoside derivatives. On the other hand, the predominant hydroxycinnamic acids have been identified as 3CQAc, 5CQAc, 3*p*CoQAc and different sinapic acid derivatives. Similar phenolic profile has already been characterized in other *Brassica* vegetables such as broccoli, turnips, kale and tronchuda cabbage (Vallejo et al., 2004; Ferreres et al., 2006; Francisco et al., 2009; Velasco et al., 2011).

Quantification of the major *B. oleracea* phenolic compounds revealed that these secondary metabolites varied greatly in concentration among lines in the DH mapping population in the three different plant organs under study. Distributions of individual and sums of phenolics were in most of cases transgressive. Therefore, new combination of additive alleles or epistatic interactions among loci for phenolics accumulation could be the cause to exceed the parental phenotypes. These types of segregation have been described before in the same DH population for GLS compounds (Sotelo et al., 2014b), and in other *B. oleracea* mapping population in a flavonoid genomic study (Lee et al., 2014).

### Phenolic composition differs across plant organs

The accumulation of some kind of phenolics was distributed in an organ-dependent manner. This different metabolic composition among organs may depend on *de novo* biosynthesis and catabolism or can also result from re-allocation of compounds. The ability to synthesize different phenolic compounds during plant developmental stages seems to be related with specific needs, allowing plants to cope with the constantly changing environmental challenges over their life cycle, thus obtaining protective adaptation to environmental stresses.

In this respect, the high diversity in the profile of flavonoids found in leaves and flower buds is not surprising. In each plant organ more than 20 structurally different flavonoids were found. On the other hand, seeds had less diversity, only one flavonoid in small quantities was found. In concordance with that, it has been described that flavonoid content and antioxidant activity in *Brassica* crops might differ completely form young to mature leaves as well as between external to internal leaves (Ferreres et al., 2006; Soengas et al., 2012). This particular accumulation of flavonoids suggests specialized functions in different stages of the plant development. It has been reported that flavonoids could have a role as internal physiological regulators or chemical messengers within the plant (Buer et al., 2010). Quercetin and kaempferol flavonoids can act through a specific receptor (NPA) in the plant cell plasma membrane blocking the polar auxin transport which could influence plant architecture (Brown et al., 2001; Buer and Muday, 2004). Besides, flavonoids have different roles in plant defense against pathogens, herbivores, and stress-related oxidative pressure (Buer et al., 2010). Therefore, any plant organ or temporal variation in the quality and quantity of flavonoids may have a role in plant organ differentiation, development, defense and adaptation.

In seeds, total phenolics content was mainly represented by only one hydroxycinnamic acid, a synapoyl derivative that was exclusively found in this plant organ. Hydroxycinnamic acids are precursors of lignin biosynthesis, important in the first plant stages to rigidifying cell walls and rendering them impermeable to water. Seeds of many species of *Brassica* genus accumulate high amount of sinapic acid derivaties such as *O*-sinapoylcholine (sinapine) and synapoyl gentiobiosides as the predominating phenolic compounds (Milkowski et al., 2004). During seed germination these compounds are transformed into sinapoylmalate which is accumulated in vacuoles of the leaf epidermal cell layer serving as UV screen. This could explain why seeds showed higher concentration of these hydroxycinnamic acids. So far, the biological role of synapoyl derivatives accumulation during seed development has not been elucidated, although it has been shown that overexpression of genes involved in sinapine metabolism influence the metabolism, morphology and physiology of developing seedlings (Clauß et al., 2011).

### Organ-specific QTLs and phenylpropanoid pathway candidate genes

In this study, we found 79 QTLs controlling phenolic content at three different organs. As it was expected, most of the detected QTLs were found in leaves and flower buds as they showed the highest variability. The meta-analysis reduced the total QTL number of independent consensus QTLs to 33 and they were spread all over the nine linkage groups. Comparative analysis of the consensus QTLs among plant organs revealed that only two of the consensus QTLs were located in the same genomic region for the three organs under study; while 23 of them were found within a single plant organ. Thus, most of the studied compounds had organ-specific genomic control, probably due to a selective expression of distinct fractions of the genome in response to developmental and environmental cues.

It has been shown that organ type is the most important factor in determining differentially expressed genes in *A. thaliana* (Schmid et al., 2005). The consensus QTL-9.3 seems to control most of the phenolics traits only in leaves. *In silico* mapping approaches located two candidate genes in this region, a putative HCT and a CCR (Table S1). These genes are in the core pathway of the phenylpropanoid metabolism influencing the accumulation of flavonoids, chlorogenic and sinapic acids derivatives in *A. thaliana* (Fraser and Chapple, 2011) (Figure 1). In flower buds, the consensus QTL-6.3, QTL-7.1, and QTL-9.4 regulated the accumulation of different traits including total phenolic content, individual flavonoids and hydroxycinnamic acids content. Two putative UDP-glucosyltransferases seem to be involved on the phenotypic variation related with QTL-7.1. In seeds, the QTL-8.4 regulated the content of the major hydroxycinnamic acids exclusively in this plant organ; unfortunately it was not possible to do *in silico* mapping in this region. Our findings, suggest a different gene regulation in the accumulation of phenolic compounds depending on the developmental stage of the plant or environmental signals. Moreover, this could explain the different metabolic profiles of phenolics accumulation among organs and will help to elaborate the genetic mechanism of phenolic biosynthesis in *B. oleracea*.

Only in two genomic regions, at C7 and C9 linkage groups, co-located consensus QTLs were found for leaves, flower buds and seeds (QTL-7.3 and QTL-9.5). They were related with up to seven traits including individual flavonoids, hydroxycinnamics as well as total phenolic compounds and explained between 6 and 20% of the phenotypic variation for those traits. This suggests that the genes that underlie these consensus QTLs could be involved in the core pathway of phenolics biosynthesis. By *in silico* analysis within the *B. oleracea* genome it was proposed a candidate gene (*Bol043270*) that could explain the phenotypic variation of the QTL-7.3 (Table S1). This gene has *O*-methyltransferase activity and codifies for a putative CCoAMT. In *A. thaliana*, this enzyme catalyzes the methylation of caffeoyl-CoA to feruloyl-CoA (*in vitro* and *in vivo*) and 5-hydroxyferuloyl-CoA to sinapoyl-CoA (at least *in vitro*) and, together with COMT, are involved in redundant functions for lignin, flavonoids and sinapoyl malate biosynthesis (Do et al., 2007). Disrupting the expression of both CCoAOMT and COMT dramatically reduces G and S lignin and 3-*O*-methoxylated soluble metabolites in *A. thaliana* indicating a main role of those enzymes in the phenylpropanoid pathway (Raes et al., 2003). The other consensus QTL shared by the three organs under study, the QTL-9.5, mapped to regions previously identified to contain a major QTL for individual and total GLS content in the same *B. oleracea* population (Sotelo et al., 2014b). Unfortunately it was not possible to use the *in silico* approach to localize candidate genes in this region. The fact that a single QTL affects the content of different secondary metabolites suggests that there may be a regulatory loci underlying the QTL or that there is a tight linkage of distinct genes involved in those metabolites accumulation. Co-localization of different QTLs might also be a first indication that some loci have a pleiotropic effect, due to a common mechanistic basis. Although GLSs and phenylpropanoids are synthesized through distinct biosynthetic pathways and have unique functions, Kim et al. (2015) showed that there is crosstalk between the two pathways. Significant contribution of the region underlying QTL-9.5 to the regulation of the level of secondary metabolites in *B. oleracea* opens the possibility for application of metabolic engineering in *Brassica* crops. However, it is important to realize that correlation between different pathways can exist, preventing the identification of a hotspot for regulation of specific metabolites. In the present study, since the markers were not uniformly distributed, large gaps appeared with low marker density on the region of the QTL-9.5 implying that more markers should be developed among these gaps and the authenticity of those common QTL for secondary metabolism should be further clarified.

Consensus QTLs were also detected for a specific kind of compounds. Some of the QTLs related with individual hydroxycinnamic acids explained a large proportion of the observed variation suggesting that hydroxycinnamics accumulation in *B. oleracea* is under the genetic control of a few additive loci. The QTL-4.3 regulated caffeoyl and *p*-coumaroyl quinic acids content in leaves and flower buds. This QTL showed the highest LOD scores and explained a large proportion of the variation for those traits (Table S3). Presence of a specific allele in this region of C4 resulted in higher levels of *p*-coumaroyl quinic acids and lower levels of caffeoyl quinic acids within both organs. In the confidence interval of this QTL we found synteny with a region of *A. thaliana* that mapped for a key gene of the phenylpropanoid pathway which codifies C3H enzyme. The C3H is a cytochrome P450 which transfers another hydroxyl group to the 3-position of the aromatic ring in *p*-coumaric acid required for the production of caffeoyl quinic acids (Figure 1). It is described as one of the main enzymes for the cinnamic intermediates in lignin biosynthesis (Rosler et al., 1997; Berner et al., 2006). The position of this QTL on the top of C4 linkage group co-localized with a previous QTL described by Sotelo et al. (2014a) related with antioxidant capacity in this *B. oleracea* population. This finding strongly supports further validation by fine mapping of C3H gene in the *B. oleracea* genome.

The hypothesis that regulation of flavonoid accumulation is a complex trait affected by many small effect alleles is also supported by the fact that at least 10 consensus QTLs were exclusively related with this kind of phenolics, being most of them also organ-specific. Moreover, most of those QTLs explained less than 10% of the phenotypic variation for a specific flavonoid trait. While over 60 genes have now been characterized to be involved in flavonoid metabolism, we could not find any known genes in the flavonoid metabolic pathway by *in silico* approaches. Recently, a study on *A. thaliana* seed flavonoid QTLs carried out by Routaboul et al. (2012) had similar results meaning that most loci identified did not co-localize with any gene previously characterized in the phenylpropanoid pathway. These findings suggest that the regulation of flavonoid accumulation may involve higher order transcriptional control or other yet uncharacterized limiting step such as transporter activity.

Since plant phenolics can modulate essential biological processes in the plant and are involved in a variety of defense response, a better understanding of the genomic regions that regulate phenol accumulation in *Brassica* vegetables within plant organs would be useful in terms of plant physiological ecology. The availability of the whole *B. oleracea* genome sequence together with possible comparative alignment with the related model species *A. thaliana* will facilitate fine mapping and cloning of candidate genes underlying the desired QTL. In addition, this information is also of great importance to both consumers and producers, as it can accelerate breeding programs to enhance plant defense and health protecting capacity of *Brassica* crops while decreasing the antinutritional properties of others.

## Author contributions

Conception and design of the work: MF, PV, and PS; acquisition, analysis, and data interpretation: MF, MA, FF, DM, PV, and PS; wrote the paper: MF and PS.

## Funding

Research supported by the Spanish Ministry of Economy and Competitiveness through the project AGL2012-35539 supported in part by the European Union (FEDER funds).

### Conflict of interest statement

The authors declare that the research was conducted in the absence of any commercial or financial relationships that could be construed as a potential conflict of interest.
