# Supplementary material for: Organ-Specific Quantitative Genetics and Candidate Genes of Phenylpropanoid Metabolism in Brassica oleracea
Source: Front Plant Sci. 2016 Jan 28;6:1240. doi: 10.3389/fpls.2015.01240 (PMC4729930; doi:10.3389/fpls.2015.01240)
Supplement: Supplementary file 1 [file Table1.DOCX]

Table S1. List of *B. oleracea* candidate genes related to phenylpropanoid pathway in those regions corresponding to the confidence interval of the described QTL.

| *B.oleracea* Genes | KEGG Orthology Entry | EC no. | Linkage group^1^ | Consensus QTL | Coordinate range (bp) | Description |
| --- | --- | --- | --- | --- | --- | --- |
| [Bol039613](http://www.ocri-genomics.org/cgi-bin/bolbase/gene_detail.cgi?locus=Bol039613) | [K00588](http://www.genome.jp/dbget-bin/www_bget?ko:K00588) | [2.1.1.104](http://www.genome.jp/dbget-bin/www_bget?ec:2.1.1.104) | 1 | QTL-1.2 | [12529933 : 12530907](http://119.97.203.210/cgi-bin/gb2/gbrowse/bolbase_chr/?name=C01:12529933..12530907) | caffeoyl-CoA O-methyltransferase |
| [Bol039614](http://www.ocri-genomics.org/cgi-bin/bolbase/gene_detail.cgi?locus=Bol039614) | [K00588](http://www.genome.jp/dbget-bin/www_bget?ko:K00588) | [2.1.1.104](http://www.genome.jp/dbget-bin/www_bget?ec:2.1.1.104) | 1 | QTL-1.2 | [12535786 : 12536828](http://119.97.203.210/cgi-bin/gb2/gbrowse/bolbase_chr/?name=C01:12535786..12536828) | probable caffeoyl-CoA O-methyltransferase |
| [Bol029008](http://www.ocri-genomics.org/cgi-bin/bolbase/gene_detail.cgi?locus=Bol029008) | [K09755](http://www.genome.jp/dbget-bin/www_bget?ko:K09755) | [1.14.-.-](http://www.genome.jp/dbget-bin/www_bget?ec:1.14.-.-) | 1 | QTL-1.2 | [1388702 : 1391218](http://119.97.203.210/cgi-bin/gb2/gbrowse/bolbase_chr/?name=C01:1388702..1391218) | ferulate-5-hydroxylase |
| [Bol021190](http://www.ocri-genomics.org/cgi-bin/bolbase/gene_detail.cgi?locus=Bol021190) | [K09753](http://www.genome.jp/dbget-bin/www_bget?ko:K09753) | [1.2.1.44](http://www.genome.jp/dbget-bin/www_bget?ec:1.2.1.44) | 2 | QTL-2.1 | [3286649 : 3290243](http://119.97.203.210/cgi-bin/gb2/gbrowse/bolbase_chr/?name=C02:3286649..3290243) | cinnamoyl-CoA reductase |
| [Bol010487](http://www.ocri-genomics.org/cgi-bin/bolbase/gene_detail.cgi?locus=Bol010487) | [K13065](http://www.genome.jp/dbget-bin/www_bget?ko:K13065) | [2.3.1.133](http://www.genome.jp/dbget-bin/www_bget?ec:2.3.1.133) | 2 | QTL-2.3 | [11815201 : 11816304](http://119.97.203.210/cgi-bin/gb2/gbrowse/bolbase_chr/?name=C02:11815201..11816304) | anthranilate N-hydroxycinnamoyl/benzoyltransferase-like protein OS=Arabidopsis thaliana |
| [Bol028633](http://www.ocri-genomics.org/cgi-bin/bolbase/gene_detail.cgi?locus=Bol028633) | [K09753](http://www.genome.jp/dbget-bin/www_bget?ko:K09753) | [1.2.1.44](http://www.genome.jp/dbget-bin/www_bget?ec:1.2.1.44) | 2 | QTL-2.3 | [12862976 : 12864499](http://119.97.203.210/cgi-bin/gb2/gbrowse/bolbase_chr/?name=C02:12862976..12864499) | cinnamoyl-CoA reductase |
| [Bol011801](http://www.ocri-genomics.org/cgi-bin/bolbase/gene_detail.cgi?locus=Bol011801) | [K09753](http://www.genome.jp/dbget-bin/www_bget?ko:K09753) | [1.2.1.44](http://www.genome.jp/dbget-bin/www_bget?ec:1.2.1.44) | 2 | QTL-2.4 | [21733132 : 21734577](http://119.97.203.210/cgi-bin/gb2/gbrowse/bolbase_chr/?name=C02:21733132..21734577) | cinnamoyl-CoA reductase |
| [Bol035959](http://www.ocri-genomics.org/cgi-bin/bolbase/gene_detail.cgi?locus=Bol035959) | [K13065](http://www.genome.jp/dbget-bin/www_bget?ko:K13065) | [2.3.1.133](http://www.genome.jp/dbget-bin/www_bget?ec:2.3.1.133) | 2 | QTL-2.4 | [26307641 : 26310831](http://119.97.203.210/cgi-bin/gb2/gbrowse/bolbase_chr/?name=C02:26307641..26310831) | shikimate O-hydroxycinnamoyltransferase |
| [Bol015141](http://www.ocri-genomics.org/cgi-bin/bolbase/gene_detail.cgi?locus=Bol015141) | [K09753](http://www.genome.jp/dbget-bin/www_bget?ko:K09753) | [1.2.1.44](http://www.genome.jp/dbget-bin/www_bget?ec:1.2.1.44) | 2 | QTL-2.4 | [35116465 : 35118192](http://119.97.203.210/cgi-bin/gb2/gbrowse/bolbase_chr/?name=C02:35116465..35118192) | cinnamoyl-CoA reductase |
| [Bol031223](http://www.ocri-genomics.org/cgi-bin/bolbase/gene_detail.cgi?locus=Bol031223) | [K13065](http://www.genome.jp/dbget-bin/www_bget?ko:K13065) | [2.3.1.133](http://www.genome.jp/dbget-bin/www_bget?ec:2.3.1.133) | 2 | QTL-2.4 | [39293435 : 39294127](http://119.97.203.210/cgi-bin/gb2/gbrowse/bolbase_chr/?name=C02:39293435..39294127) | shikimate O-hydroxycinnamoyltransferase |
| [Bol031224](http://www.ocri-genomics.org/cgi-bin/bolbase/gene_detail.cgi?locus=Bol031224) | [K13065](http://www.genome.jp/dbget-bin/www_bget?ko:K13065) | [2.3.1.133](http://www.genome.jp/dbget-bin/www_bget?ec:2.3.1.133) | 2 | QTL-2.4 | [39294134 : 39294793](http://119.97.203.210/cgi-bin/gb2/gbrowse/bolbase_chr/?name=C02:39294134..39294793) | shikimate O-hydroxycinnamoyltransferase |
| [Bol042450](http://www.ocri-genomics.org/cgi-bin/bolbase/gene_detail.cgi?locus=Bol042450) | [K12355](http://www.genome.jp/dbget-bin/www_bget?ko:K12355) | [1.2.1.68](http://www.genome.jp/dbget-bin/www_bget?ec:1.2.1.68) | 3 | QTL-3.3 | [25944756 : 25948548](http://119.97.203.210/cgi-bin/gb2/gbrowse/bolbase_chr/?name=C03:25944756..25948548) | coniferyl-aldehyde dehydrogenase |
| [Bol037689](http://www.ocri-genomics.org/cgi-bin/bolbase/gene_detail.cgi?locus=Bol037689) | [K10775](http://www.genome.jp/dbget-bin/www_bget?ko:K10775) | [4.3.1.24](http://www.genome.jp/dbget-bin/www_bget?ec:4.3.1.24) | 4 | QTL-4.2 | [37110551 : 37113084](http://119.97.203.210/cgi-bin/gb2/gbrowse/bolbase_chr/?name=C04:37110551..37113084) | phenylalanine ammonia-lyase |
| [Bol038332](http://www.ocri-genomics.org/cgi-bin/bolbase/gene_detail.cgi?locus=Bol038332) | [K12356](http://www.genome.jp/dbget-bin/www_bget?ko:K12356) | [2.4.1.111](http://www.genome.jp/dbget-bin/www_bget?ec:2.4.1.111) | 5 | QTL-5.3 | [19294113 : 19294895](http://119.97.203.210/cgi-bin/gb2/gbrowse/bolbase_chr/?name=C05:19294113..19294895) | anthocyanidin 3-O-glucosyltransferase 6 |
| [Bol038351](http://www.ocri-genomics.org/cgi-bin/bolbase/gene_detail.cgi?locus=Bol038351) | [K13068](http://www.genome.jp/dbget-bin/www_bget?ko:K13068) | [2.4.1.120](http://www.genome.jp/dbget-bin/www_bget?ec:2.4.1.120) | 5 | QTL-5.3 | [19503909 : 19504577](http://119.97.203.210/cgi-bin/gb2/gbrowse/bolbase_chr/?name=C05:19503909..19504577) | UDP-glucose:sinapate glucosyltransferase |
| [Bol038385](http://www.ocri-genomics.org/cgi-bin/bolbase/gene_detail.cgi?locus=Bol038385) | [K01904](http://www.genome.jp/dbget-bin/www_bget?ko:K01904) | [6.2.1.12](http://www.genome.jp/dbget-bin/www_bget?ec:6.2.1.12) | 5 | QTL-5.3 | [19959988 : 19960618](http://119.97.203.210/cgi-bin/gb2/gbrowse/bolbase_chr/?name=C05:19959988..19960618) | 4-coumarate--CoA ligase |
| [Bol038386](http://www.ocri-genomics.org/cgi-bin/bolbase/gene_detail.cgi?locus=Bol038386) | [K01904](http://www.genome.jp/dbget-bin/www_bget?ko:K01904) | [6.2.1.12](http://www.genome.jp/dbget-bin/www_bget?ec:6.2.1.12) | 5 | QTL-5.3 | [19972174 : 19973519](http://119.97.203.210/cgi-bin/gb2/gbrowse/bolbase_chr/?name=C05:19972174..19973519) | 4-coumarate--CoA ligase |
| [Bol038387](http://www.ocri-genomics.org/cgi-bin/bolbase/gene_detail.cgi?locus=Bol038387) | [K01904](http://www.genome.jp/dbget-bin/www_bget?ko:K01904) | [6.2.1.12](http://www.genome.jp/dbget-bin/www_bget?ec:6.2.1.12) | 5 | QTL-5.3 | [20005776 : 20015923](http://119.97.203.210/cgi-bin/gb2/gbrowse/bolbase_chr/?name=C05:20005776..20015923) | 4-coumarate--CoA ligase |
| *B.oleracea* Genes | KEGG Orthology Entry | EC no. | Linkage group^1^ | Consensus QTL | Coordinate range (bp) | Description |
| [Bol038389](http://www.ocri-genomics.org/cgi-bin/bolbase/gene_detail.cgi?locus=Bol038389) | [K01904](http://www.genome.jp/dbget-bin/www_bget?ko:K01904) | [6.2.1.12](http://www.genome.jp/dbget-bin/www_bget?ec:6.2.1.12) | 5 | QTL-5.3 | [20103192 : 20107888](http://119.97.203.210/cgi-bin/gb2/gbrowse/bolbase_chr/?name=C05:20103192..20107888) | 4-coumarate--CoA ligase |
| [Bol018102](http://www.ocri-genomics.org/cgi-bin/bolbase/gene_detail.cgi?locus=Bol018102) | [K00083](http://www.genome.jp/dbget-bin/www_bget?ko:K00083) | [1.1.1.195](http://www.genome.jp/dbget-bin/www_bget?ec:1.1.1.195) | 5 | QTL-5.3 | [23398700 : 23400580](http://119.97.203.210/cgi-bin/gb2/gbrowse/bolbase_chr/?name=C05:23398700..23400580) | cinnamyl-alcohol dehydrogenase |
| [Bol016957](http://www.ocri-genomics.org/cgi-bin/bolbase/gene_detail.cgi?locus=Bol016957) | [K12356](http://www.genome.jp/dbget-bin/www_bget?ko:K12356) | [2.4.1.111](http://www.genome.jp/dbget-bin/www_bget?ec:2.4.1.111) | 7 | QTL-7.1 | [37730198 : 37731670](http://119.97.203.210/cgi-bin/gb2/gbrowse/bolbase_chr/?name=C07:37730198..37731670) | anthocyanidin 3-O-glucosyltransferase 5 OS=Manihot esculenta |
| [Bol016956](http://www.ocri-genomics.org/cgi-bin/bolbase/gene_detail.cgi?locus=Bol016956) | [K12356](http://www.genome.jp/dbget-bin/www_bget?ko:K12356) | [2.4.1.111](http://www.genome.jp/dbget-bin/www_bget?ec:2.4.1.111) | 7 | QTL-7.1 | [37736992 : 37738452](http://119.97.203.210/cgi-bin/gb2/gbrowse/bolbase_chr/?name=C07:37736992..37738452) | anthocyanidin 3-O-glucosyltransferase 5 OS=Manihot esculenta |
| [Bol043270](http://www.ocri-genomics.org/cgi-bin/bolbase/gene_detail.cgi?locus=Bol043270) | [K00588](http://www.genome.jp/dbget-bin/www_bget?ko:K00588) | [2.1.1.104](http://www.genome.jp/dbget-bin/www_bget?ec:2.1.1.104) | 7 | QTL-7.3 | [21266084 : 21268666](http://119.97.203.210/cgi-bin/gb2/gbrowse/bolbase_chr/?name=C07:21266084..21268666) | caffeoyl-CoA O-methyltransferase |
| [Bol044581](http://www.ocri-genomics.org/cgi-bin/bolbase/gene_detail.cgi?locus=Bol044581) | [K00588](http://www.genome.jp/dbget-bin/www_bget?ko:K00588) | [2.1.1.104](http://www.genome.jp/dbget-bin/www_bget?ec:2.1.1.104) | 8 | QTL-8.3 | [33093642 : 33095204](http://119.97.203.210/cgi-bin/gb2/gbrowse/bolbase_chr/?name=C08:33093642..33095204) | caffeoyl-CoA O-methyltransferase |
| [Bol044759](http://www.ocri-genomics.org/cgi-bin/bolbase/gene_detail.cgi?locus=Bol044759) | [K01188](http://www.genome.jp/dbget-bin/www_bget?ko:K01188) | [3.2.1.21](http://www.genome.jp/dbget-bin/www_bget?ec:3.2.1.21) | 8 | QTL-8.3 | [35484628 : 35488994](http://119.97.203.210/cgi-bin/gb2/gbrowse/bolbase_chr/?name=C08:35484628..35488994) | thioglucosidase |
| [Bol044760](http://www.ocri-genomics.org/cgi-bin/bolbase/gene_detail.cgi?locus=Bol044760) | [K01188](http://www.genome.jp/dbget-bin/www_bget?ko:K01188) | [3.2.1.21](http://www.genome.jp/dbget-bin/www_bget?ec:3.2.1.21) | 8 | QTL-8.3 | [35490959 : 35493786](http://119.97.203.210/cgi-bin/gb2/gbrowse/bolbase_chr/?name=C08:35490959..35493786) | thioglucosidase |
| [Bol044768](http://www.ocri-genomics.org/cgi-bin/bolbase/gene_detail.cgi?locus=Bol044768) | [K00083](http://www.genome.jp/dbget-bin/www_bget?ko:K00083) | [1.1.1.195](http://www.genome.jp/dbget-bin/www_bget?ec:1.1.1.195) | 8 | QTL-8.3 | [35603130 : 35605051](http://119.97.203.210/cgi-bin/gb2/gbrowse/bolbase_chr/?name=C08:35603130..35605051) | cinnamyl-alcohol dehydrogenase |
| [Bol038890](http://www.ocri-genomics.org/cgi-bin/bolbase/gene_detail.cgi?locus=Bol038890) | [K13065](http://www.genome.jp/dbget-bin/www_bget?ko:K13065) | [2.3.1.133](http://www.genome.jp/dbget-bin/www_bget?ec:2.3.1.133) | 9 | QTL-9.3 | [23051687 : 23053108](http://119.97.203.210/cgi-bin/gb2/gbrowse/bolbase_chr/?name=C09:23051687..23053108) | uncharacterized acetyltransferase At3g50280 |
| [Bol016918](http://www.ocri-genomics.org/cgi-bin/bolbase/gene_detail.cgi?locus=Bol016918) | [K09753](http://www.genome.jp/dbget-bin/www_bget?ko:K09753) | [1.2.1.44](http://www.genome.jp/dbget-bin/www_bget?ec:1.2.1.44) | 9 | QTL-9.3 | [25988009 : 25989364](http://119.97.203.210/cgi-bin/gb2/gbrowse/bolbase_chr/?name=C09:25988009..25989364) | cinnamoyl-CoA reductase |
| [Bol016898](http://www.ocri-genomics.org/cgi-bin/bolbase/gene_detail.cgi?locus=Bol016898) | [K09753](http://www.genome.jp/dbget-bin/www_bget?ko:K09753) | [1.2.1.44](http://www.genome.jp/dbget-bin/www_bget?ec:1.2.1.44) | 9 | QTL-9.3 | [26096456 : 26097828](http://119.97.203.210/cgi-bin/gb2/gbrowse/bolbase_chr/?name=C09:26096456..26097828) | dihydroflavonol 4-reductase-like OS=Arabidopsis thaliana |

^1^ Linkage group number are related to *B. oleracea* chromosomes 1 to 9.
