# Supplementary material for: Organ-Specific Quantitative Genetics and Candidate Genes of Phenylpropanoid Metabolism in Brassica oleracea
Source: Front Plant Sci. 2016 Jan 28;6:1240. doi: 10.3389/fpls.2015.01240 (PMC4729930; doi:10.3389/fpls.2015.01240)
Supplement: Supplementary file 2 [file Table2.DOCX]

Table S2. List of *Arabidopsis thaliana* genes related to phenylpropanoid pathway, obtained from TAIR (The Arabidopsis Information Resource)

| Gene name | Linkage group | Coordinate range (bp) | Description |
| --- | --- | --- | --- |
| AT1G02050 | 1 | 359117-360518 | chalcone and stilbene synthase family protein |
| AT1G15670 | 1 | 5389952-5391490 | galactose oxidase/kelch repeat superfamily protein |
| AT1G17010 | 1 | 5817567-5819352 | 2-oxoglutarate (2OG) and Fe(II)-dependent oxygenase superfamily protein |
| AT1G20480 | 1 | 7094833-7097114 | AMP-dependent synthetase and ligase family protein |
| AT1G20490 | 1 | 7097293-7099695 | AMP-dependent synthetase and ligase family protein |
| AT1G20500 | 1 | 7100502-7102915 | AMP-dependent synthetase and ligase family protein |
| AT1G20510 | 1 | 7103454-7105881 | OPC-8:0 CoA ligase1 |
| AT1G22640 | 1 | 8006195-8007428 | myb domain protein 3 |
| AT1G25460 | 1 | 8942811-8944244 | NAD(P)-binding Rossmann-fold superfamily protein |
| AT1G32640 | 1 | 11798810-11800988 | basic helix-loop-helix (bHLH) DNA-binding family protein |
| AT1G34790 | 1 | 12763902-12765638 | C2H2 and C2HC zinc fingers superfamily protein |
| AT1G49390 | 1 | 18279600-18280978 | 2-oxoglutarate (2OG) and Fe(II)-dependent oxygenase superfamily protein |
| AT1G51680 | 1 | 19158752-19161552 | 4-coumarate:CoA ligase 1 |
| AT1G61720 | 1 | 22791168-22792800 | NAD(P)-binding Rossmann-fold superfamily protein |
| AT1G65060 | 1 | 24167202-24171502 | 4-coumarate:CoA ligase 3 |
| Gene name | Linkage group | Coordinate range (bp) | Description |
| AT1G80440 | 1 | 30241607-30243071 | galactose oxidase/kelch repeat superfamily protein |
| AT2G03760 | 2 | 1149337-1150663 | sulphotransferase 12 |
| AT2G03770 | 2 | 1150871-1151845 | P-loop containing nucleoside triphosphate hydrolases superfamily protein |
| AT2G26170 | 2 | 11140809-11143360 | cytochrome P450, family 711, subfamily A, polypeptide 1 |
| AT2G30490 | 2 | 12993663-12995770 | cinnamate-4-hydroxylase |
| AT2G37040 | 2 | 15557376-15560363 | PHE ammonia lyase 1 |
| AT2G40890 | 2 | 17058053-17060652 | cytochrome P450, family 98, subfamily A, polypeptide 3 |
| AT2G47460 | 2 | 19476337-19479478 | myb domain protein 12 |
| AT2G48110 | 2 | 19673293-19679711 | reduced epidermal fluorescence 4 |
| AT3G10310 | 3 | 3190001-3195005 | P-loop nucleoside triphosphate hydrolases superfamily protein with CH (Calponin Homology) domain |
| AT3G13610 | 3 | 4449374-4450811 | 2-oxoglutarate (2OG) and Fe(II)-dependent oxygenase superfamily protein |
| AT3G19010 | 3 | 6556197-6557938 | 2-oxoglutarate (2OG) and Fe(II)-dependent oxygenase superfamily protein |
| AT3G21230 | 3 | 7448040-7452000 | 4-coumarate:CoA ligase 5 |
| AT3G21240 | 3 | 7454269-7457379 | 4-coumarate:CoA ligase 2 |
| AT3G21560 | 3 | 7595806-7597577 | UDP-Glycosyltransferase superfamily protein |
| AT3G21750 | 3 | 7664345-7666195 | UDP-glucosyl transferase 71B1 |
| AT3G23590 | 3 | 8467449-8473627 | REF4-related 1 |
| Gene name | Linkage group | Coordinate range (bp) | Description |
| AT3G24503 | 3 | 8919560-8923073 | aldehyde dehydrogenase 2C4 |
| AT3G29670 | 3 | 11527872-11529350 | HXXXD-type acyl-transferase family protein |
| AT3G45070 | 3 | 16486283-16487349 | P-loop containing nucleoside triphosphate hydrolases superfamily protein |
| AT3G51240 | 3 | 19025192-19026872 | flavanone 3-hydroxylase |
| AT3G53260 | 3 | 19744051-19746780 | phenylalanine ammonia-lyase 2 |
| AT3G53480 | 3 | 19825307-19831797 | pleiotropic drug resistance 9 |
| AT3G59940 | 3 | 22142900-22144439 | galactose oxidase/kelch repeat superfamily protein |
| AT3G62980 | 3 | 23273116-23276375 | F-box/RNI-like superfamily protein |
| AT4G00040 | 4 | 14627-16079 | chalcone and stilbene synthase family protein |
| AT4G08770 | 4 | 5598115-5600312 | peroxidase superfamily protein |
| AT4G09820 | 4 | 6182023-6186493 | basic helix-loop-helix (bHLH) DNA-binding superfamily protein |
| AT4G14090 | 4 | 8122188-8123835 | UDP-Glycosyltransferase superfamily protein |
| AT4G15480 | 4 | 8848801-8850516 | UDP-Glycosyltransferase superfamily protein |
| AT4G15490 | 4 | 8852698-8854545 | UDP-Glycosyltransferase superfamily protein |
| AT4G15500 | 4 | 8856988-8858572 | UDP-Glycosyltransferase superfamily protein |
| AT4G16330 | 4 | 9226122-9227953 | 2-oxoglutarate (2OG) and Fe(II)-dependent oxygenase superfamily protein |
| AT4G16770 | 4 | 9434393-9437172 | 2-oxoglutarate (2OG) and Fe(II)-dependent oxygenase superfamily protein |
| AT4G24520 | 4 | 12662841-12667161 | P450 reductase 1 |
| Gene name | Linkage group | Coordinate range (bp) | Description |
| AT4G25300 | 4 | 12945173-12946778 | 2-oxoglutarate (2OG) and Fe(II)-dependent oxygenase superfamily protein |
| AT4G25310 | 4 | 12949664-12951234 | 2-oxoglutarate (2OG) and Fe(II)-dependent oxygenase superfamily protein |
| AT4G25640 | 4 | 13076444-13079048 | detoxifying efflux carrier 35 |
| AT4G30210 | 4 | 14796764-14800922 | P450 reductase 2 |
| AT4G34850 | 4 | 16608313-16609829 | chalcone and stilbene synthase family protein |
| AT5G04230 | 5 | 1160634-1163898 | phenyl alanine ammonia-lyase 3 |
| AT5G04410 | 5 | 1243758-1246684 | NAC domain containing protein 2 |
| AT5G07480 | 5 | 2367167-2369554 | KAR-UP oxidoreductase 1 |
| AT5G07990 | 5 | 2560394-2563109 | cytochrome P450 superfamily protein |
| AT5G26310 | 5 | 9234685-9236385 | UDP-Glycosyltransferase superfamily protein |
| AT5G39050 | 5 | 15634586-15636065 | HXXXD-type acyl-transferase family protein |
| AT5G48930 | 5 | 19836299-19838162 | hydroxycinnamoyl-CoA shikimate/quinate hydroxycinnamoyl transferase |
| AT5G66690 | 5 | 26625080-26626793 | UDP-Glycosyltransferase superfamily protein |
