# Supplementary material for: Organ-Specific Quantitative Genetics and Candidate Genes of Phenylpropanoid Metabolism in Brassica oleracea
Source: Front Plant Sci. 2016 Jan 28;6:1240. doi: 10.3389/fpls.2015.01240 (PMC4729930; doi:10.3389/fpls.2015.01240)
Supplement: Supplementary file 3 [file Table3.DOCX]

Table S3. List of quantitative trait loci (QTL) for phenolics traits in leaves, flower buds and seeds of the BolTBDH mapping population.

| Plant organ | Trait | Linkage group^1^ | Peak position | Confidence interval (cM)^2^ | Left marker | Right marker | LOD score | Add^3^ | Frequency % | R^2^%^4^ |
| --- | --- | --- | --- | --- | --- | --- | --- | --- | --- | --- |
| Leaves | 3CQAc | 4 | 15 | 10-18 | pW149cD | pW205aH | 18.39 | -2.48 | 77 | 46.05 |
|  |  | 5 | 29 | 20-34 | pX140aX | pW247aE | 4.48 | 0.94 | 47.9 | 10.26 |
|  |  | 7 | 81 | 78-85 | pW128aH | CHS28aX | 3.34 | -0.76 | 12.2 | 7.28 |
|  |  | 9 | 20 | 18-23 | fito163 | pW203dX | 4.17 | -0.77 | 41.1 | 7.79 |
| Flower buds | 5CQAc | 4 | 12 | 7-18 | pX103dD | pW149cD | 14.00 | -1.73 | 72 | 36.79 |
| Leaves | 3pCoQAc | 4 | 14 | 9-16 | pX103dD | pW149cD | 18.76 | 2.27 | 99.7 | 39.45 |
|  |  | 8 | 3 | 0-10 | pX103cD | fito040d | 3.17 | 1.88 | 31.8 | 8.03 |
| Flower buds | 3pCoQAc | 7 | 77 | 76-79 | pW162cH | pX126aX | 4.99 | -1.00 | 89.7 | 14.06 |
| Flower buds | Caff. derv1 | 2 | 62 | 60-64 | pX133aX | pW161aX | 2.75 | -0.62 | 17.6 | 6.58 |
|  |  | 9 | 12 | 9-14 | pX140bX | pX140dX | 3.41 | 0.80 | 59.5 | 9.74 |
| Leaves | F1 | 2 | 42 | 27-53 | FLC2bH | pW189bX | 2.97 | 0.45 | 15.5 | 7.15 |
|  |  | 2 | 88 | 82-95 | fito019 | fito375 | 3.12 | -0.51 | 21.8 | 10.25 |
|  |  | 5 | 28 | 22-41 | pX140aX | pW247aE | 4.07 | 0.44 | 57.9 | 8.4 |
|  |  | 6 | 48 | 33--54 | pW208aE | pX144bE | 3.34 | 0.46 | 50.4 | 9.35 |
|  |  | 7 | 79 | 77-81 | fito398 | pW128aH | 6.29 | -0.58 | 93.7 | 15.11 |
|  |  | 9 | 57 | 43-71 | pW108gH | fito016 | 3.50 | 0.48 | 37.1 | 9.93 |
| Leaves | F5 | 2 | 49 | 37-54 | pW189bX | fito081a | 3.11 | 0.20 | 64.6 | 10.6 |
| Plant organ | Trait | Linkage group^1^ | Peak position | Confidence interval (cM) ^2^ | Left marker | Right marker | LOD score | Add^3^ | Frequency % | R^2^%^4^ |
|  |  |  |  |  |  |  |  |  |  |  |
|  |  | 7 | 53 | 43-60 | BRMS042 | pW108aH | 3.31 | 0.22 | 66.7 | 11.53 |
| Leaves | F7 | 6 | 54 | 41-57 | pX144bE | fito204d | 2.67 | 0.21 | 30.4 | 8.44 |
| Leaves | F8 | 2 | 57 | 50-61 | pW189bX | fito081a | 4.86 | 0.20 | 82.2 | 15.86 |
|  |  | 7 | 78 | 77-81 | pX126aX | fito398 | 2.92 | -0.27 | 24.9 | 6.23 |
| Flower buds | F11 | 7 | 58 | 45-61 | pW108aH | fito088b | 2.93 | 0.17 | 12.6 | 2.67 |
| Seeds | F11 | 3 | 27 | 26-33 | fito066 | fito262 | 3.07 | 0.08 | 37.6 | 7.09 |
|  |  | 7 | 81 | 79-84 | pW128aH | CHS28aX | 5.72 | 0.06 | 29.6 | 10.15 |
|  |  | 8 | 41 | 37-46 | pW130aE | fito204a | 3.09 | 0.06 | 32.7 | 10.62 |
| Flower buds | F14 | 8 | 42 | 37-46 | pW130aE | fito204a | 3.67 | 0.17 | 16.2 | 8.87 |
| Flower buds | F15 | 3 | 78 | 74-81 | pW196aH | BRMS017 | 3.78 | 0.48 | 75.3 | 11.29 |
|  |  | 7 | 41 | 33-43 | pW104aE | BRMS042 | 4.25 | 0.35 | 77.2 | 8.4 |
|  |  | 9 | 16 | 16-19 | pW174cX | pW179aH | 3.87 | 0.36 | 24.9 | 9.11 |
| Flower buds | F16 | 3 | 67 | 65-69 | pW188dE | pX149bE | 6.97 | 0.72 | 96.1 | 19.81 |
|  |  | 7 | 44 | 40-53 | BRMS042 | pW108aH | 4.22 | 0.28 | 76.3 | 11.61 |
|  |  | 8 | 45 | 42-50 | fito204a | fito018 | 3.77 | 0.36 | 30.2 | 7.82 |
| Flower buds | F18 | 6 | 61 | 58-65 | fito203a | pW134aH | 2.78 | 0.57 | 6.8 | 6.96 |
|  |  | 9 | 0 | 0-7 | FLC1aH | fito204b | 3.79 | 0.69 | 72.2 | 9.44 |
| Flower buds | F23 | 7 | 47 | 40-54 | BRMS042 | pW108aH | 6.06 | 0.23 | 88.7 | 12.67 |
| Plant organ | Trait | Linkage group^1^ | Peak position | Confidence interval (cM) ^2^ | Left marker | Right marker | LOD score | Add^3^ | Frequency % | R^2^%^4^ |
|  |  |  |  |  |  |  |  |  |  |  |
|  |  | 9 | 64 | 60-71 | fito016 | pW187bH | 3.12 | 1.10 | 14.8 | 4.53 |
| Flower buds | F28 | 5 | 64 | 62-66 | pW209aH | fito156a | 3.19 | -1.23 | 15.4 | 3.25 |
| Leaves | F25 | 2 | 90 | 81-95 | fito375 | fito034 | 3.76 | -0.40 | 78.5 | 12.15 |
|  |  |  |  |  |  |  |  |  |  |  |
| Leaves | F34 | 4 | 60 | 58-64 | fito287b | pW178bH | 3.94 | 0.27 | 24.6 | 7.87 |
| Leaves | F37 | 1 | 42 | 39-75 | fito094 | pW169cE | 2.59 | -0.54 | 14.3 | 7.14 |
|  |  | 2 | 56 | 48-61 | pW189bX | fito081a | 3.07 | 0.10 | 23.6 | 7.76 |
|  |  | 2 | 85 | 81-94 | fito019 | fito375 | 3.48 | -0.67 | 68.5 | 10.14 |
| Leaves | FG | 9 | 19 | 17-21 | fito163 | pW203dX | 3.69 | -0.30 | 63.4 | 8.97 |
| Flower buds | SA1 | 2 | 62 | 61-67 | pX133aX | pW161aX | 2.89 | -0.87 | 21.4 | 7.19 |
|  |  | 6 | 57 | 54-59 | fito204d | fito203a | 3.50 | 1.04 | 68.9 | 10.3 |
| Seeds | SA1 | 3 | 99 | 94-100 | BRMS015a | pX146dH | 3.08 | -0.09 | 46.7 | 9.16 |
|  |  | 8 | 50 | 47-53 | fito018 | pX130cD | 4.12 | -0.34 | 80.9 | 16.81 |
| Seeds | SA2 | 2 | 55 | 48-61 | pW189bX | fito081a | 4.40 | 0.27 | 31.8 | 10.41 |
| Seeds | SA2 | 8 | 19 | 18-24 | fito098b | pW170aH | 2.98 | 0.14 | 27.6 | 7.9 |
|  |  | 8 | 54 | 50-59 | fito146b | fito373c | 5.67 | 2.58 | 89.0 | 18.34 |
|  |  | 9 | 60 | 45-69 | pW108gH | fito016 | 3.26 | 8.95 | 35.1 | 6.22 |
| Leaves | SA9 | 2 | 70 | 65-72 | pW250bH | Ol13-E08 | 5.37 | -1.20 | 71.8 | 20.1 |
| Plant organ | Trait | Linkage group^1^ | Peak position | Confidence interval (cM) ^2^ | Left marker | Right marker | LOD score | Add^3^ | Frequency % | R^2^%^4^ |
|  |  |  |  |  |  |  |  |  |  |  |
|  |  | 9 | 21 | 18-23 | pW203dX | pX147iH | 5.56 | -0.83 | 57.1 | 10.86 |
| Flower buds | SA9 | 1 | 75 | 49-77 | fito098c | pW145dX | 3.04 | -0.32 | 28.5 | 7.36 |
|  |  | 3 | 56 | 53-57 | pX111aD | fito394 | 3.78 | 0.38 | 38.8 | 6.79 |
|  |  | 9 | 60 | 43-71 | pW108gH | fito016 | 2.93 | 0.40 | 61.3 | 12.16 |
| Seeds | SA9 | 2 | 61 | 60-62 | pW144aE | pX133aX | 4.80 | 1.31 | 48.9 | 6.16 |
|  |  | 4 | 57 | 56-58 | pW188cE | fito377 | 5.72 | 1.50 | 35.3 | 7.41 |
|  |  | 8 | 54 | 50-59 | fito146b | fito373c | 4.09 | 2.63 | 74.2 | 13.97 |
|  |  |  |  |  |  |  |  |  |  |  |
| Leaves | SA10 | 9 | 20 | 18-21 | fito163 | pW203dX | 9.04 | -0.97 | 77.4 | 11.23 |
| Flower buds | SA10 | 2 | 19 | 14-36 | pW241bE | FLC2bH | 5.59 | -1.20 | 24.6 | 3.68 |
|  |  | 7 | 79 | 77-81 | fito398 | pW128aH | 3.25 | -0.81 | 32.7 | 7.2 |
| Leaves | SA11 | 3 | 93 | 86-95 | FC | pW145cX | 2.83 | -0.30 | 47.4 | 7.27 |
| Flower buds | SA11 | 5 | 82 | 78-87 | pW198bH | fito294a | 3.07 | 0.28 | 32.5 | 1.32 |
|  |  | 7 | 42 | 40-51 | pW104aE | BRMS042 | 3.20 | 0.92 | 34.0 | 8.61 |
| Seeds | SA11 | 3 | 93 | 89-95 | FC | pW145cX | 10.82 | 0.34 | 99.5 | 30.74 |
|  |  | 8 | 53 | 50-58 | fito146b | fito373c | 3.11 | -1.85 | 24.5 | 10.68 |
| Flower buds | SA12 | 6 | 81 | 80-84 | fito190 | pW130aH | 5.29 | 0.32 | 96.4 | 11.39 |
|  |  | 8 | 1 | 0-10 | pX103cD | fito040d | 2.61 | -0.60 | 28.5 | 8.35 |
| Plant organ | Trait | Linkage group^1^ | Peak position | Confidence interval (cM) ^2^ | Left marker | Right marker | LOD score | Add^3^ | Frequency % | R^2^%^4^ |
|  |  |  |  |  |  |  |  |  |  |  |
| Leaves | FlavT | 9 | 22 | 18-23 | pW203dX | pX147iH | 2.80 | -0.27 | 50.1 | 15.44 |
|  |  | 9 | 52 | 42-64 | pW108gH | fito016 | 5.98 | 0.36 | 62.9 | 19.84 |
| Flower buds | FlavT | 6 | 75 | 71-79 | pX130fD | fito190 | 3.57 | 4.66 | 52.8 | 10.65 |
|  |  | 9 | 44 | 31-60 | pW108gH | fito016 | 3.15 | 5.31 | 38.9 | 11.75 |
| Flower buds | HydroxT | 6 | 75 | 70-80 | pX130fD | fito190 | 3.64 | 4.55 | 69.6 | 10.09 |
| Seeds | HydroxT | 9 | 62 | 43-71 | fito016 | pW187bH | 2.99 | 7.60 | 40.4 | 7.35 |
| Leaves | PhenolT | 9 | 21 | 19-23 | pW203dX | pX147iH | 7.96 | -0.43 | 86.6 | 9.16 |
| Flower buds | PhenolT | 6 | 75 | 71-78 | pX130fD | fito190 | 4.84 | 9.56 | 83.2 | 14.55 |
|  |  | 9 | 43 | 31-58 | pW108gH | fito016 | 3.61 | 8.78 | 42.3 | 9.96 |
| Seeds | PhenolT | 9 | 60 | 45-69 | pW108gH | fito016 | 3.18 | 8.74 | 32.3 | 6.03 |

^1^ Linkage group numbers are related to *B. oleracea* chromosomes 1 to 9.

^2^  cM, centimorgan.

^3^ Additive effects calculated as (P2-P1)/2.

^4^ R^2^ % coefficient of determination of each QTL.

See Table S4 for compounds abbreviations.
