# Supplementary material for: Organ-Specific Quantitative Genetics and Candidate Genes of Phenylpropanoid Metabolism in Brassica oleracea
Source: Front Plant Sci. 2016 Jan 28;6:1240. doi: 10.3389/fpls.2015.01240 (PMC4729930; doi:10.3389/fpls.2015.01240)
Supplement: Supplementary file 4 [file Table4.DOCX]

Table S4. Phenolic compounds identified in *Brassica oleracea.*

| **Peak** | **COMPOUND NAME** |
| --- | --- |
|  |  |
|  | **Caffeoyl quinic acids** |
| 3CQAc | 3- caffeoyl quinic acid |
| 5CQAc | 5-caffeoyl quinic acid |
| Caff. derv1 | putative caffeoyl derivative |
| Caff. derv1 | putative caffeoyl derivative |
|  | **p-coumaroyl quinic acids** |
| 4pCoQAc | 4-*p*-coumaroyl quinic acid |
| 3pCoQAc | 3-*p*-coumaroyl quinic acid |
|  | **Feruloyl and Sinapoyl acids derivatives** |
| FG | feruloyl glucoside |
| SA1 | sinapoyl glucoside |
| SA2 | putative sinapoyl derivative |
| SA3 | 1,2-disinapoyl gentiobioside |
| SA4 | 1- sinapoyl -2, 2’-dicaffeoyl gentiobioside |
| SA5 | caffeoyl, *p*-coumaroyl gentiobioside |
| SA6 | caffeoyl, feruloyl gentiobioside |
| SA7 | putative sinapoyl derivative |
| SA8 | 1-sinapoyl-2-methoxycaffeoyl gentiobioside |
| SA9 | 1,2-disinapoyl gentiobioside isomer of SA3 |
| SA10 | 1-sinapoyl-2-feruloyl gentiobioside |
| SA11 | 1, 2, 2’-tri-sinapoyl gentiobioside |
| SA12 | 1,2-disinapoyl glucoside |
| SA13 | 1, 2’-disinapoyl-2-feruloyl gentiobioside |
| SA14 | 1- sinapoyl -2-feruloyl glucoside |
| SA15 | 1-sinapoyl-2-methoxycaffeoyl gentiobioside isomer of SA8 |
|  |  |
|  |  |
|  |  |
|  |  |
| **Peak** | **COMPOUND NAME** |
|  | **Isorhamnetin derivatives** |
| F6 | isorhamnetin-3-*O-*glucoside-7-*O-*(dihydrosinapoyl)glucoside |
| F17 | isorhamnetin-3-*O*-glucoside-7,4´-*O*-glucoside |
| F18 | isorhamnetin-3-*O-*diglucoside-7-*O-*glucoside |
| F19 | isorhamnetin-3-*O-*glucoside-7-*O-*diglucoside |
| F20 | isorhamnetin-3-*O-*diglucoside-7-*O-*diglucoside |
| F44 | isorhamnetin-3-*O*-glucoside |
|  | **Kaempferol derivatives** |
| F1 | kaempferol-3-*O*-(methoxycaffeoyl)diglucoside-7-*O-*glucoside |
| F2 | kaempferol-3-*O*-(caffeoyl)diglucoside-7-*O-*glucoside |
| F5 | kaempferol-3-*O*-(sinapoyl)diglucoside-7-*O-*glucoside |
| F7 | kaempferol-3-*O*-(feruloyl)diglucoside-7-*O-*glucoside |
| F8 | kaempferol-3-*O*-(*p-*coumaroyl)diglucoside-7-*O-*glucoside |
| F10 | kaempferol-3-*O-*(sinapoyl)triglucoside-7-*O-*sophoroside |
| F11 | kaempferol-3-*O-*(caffeoyl, sinapoyl)triglucoside |
| F14 | kaempferol-3-*O-*(caffeoyl, feruloyl) diglucoside -7-*O-*diglucoside |
| F15 | kaempferol-3-*O-*diglucoside-7-*O-*glucoside |
| F16 | kaempferol-3-*O-* diglucoside -7-*O-* diglucoside |
| F21 | kaempferol-3-*O*-(methoxycaffeoyl)diglucoside |
| F23 | kaempferol-3-*O*-glucoside-7-*O*-glucoside |
| F24 | kaempferol-3-*O*-(caffeoyl)diglucoside |
| F25 | kaempferol-3-*O*-(dimethoxycaffeoyl)triglucoside-7-*O*-glucoside |
| F26 | kaempferol-3-*O*-(sinapoyl)diglucoside |
| F27 | kaempferol-3-*O*-(methoxycaffeoyl, caffeoyl)triglucoside-7-*O*-diglucoside |
| F28 | kaempferol-3-*O*- diglucoside |
| F29 | kaempferol-3-*O-*(methoxycaffeoyl, caffeoyl)triglucoside-7-*O*-glucoside isomer of F27 |
| F31 | kaempferol-3-*O*-(methoxycaffeoyl, sinapoyl)triglucoside-7-*O*-glucoside |
|  |  |
|  |  |
| **Peak** | **COMPOUND NAME** |
| F32 | kaempferol-3-*O*-(caffeoyl, sinapoyl) triglucoside-7-*O*-diglucoside |
| F33 | kaempferol-3-*O*-(feruloyl)diglucoside |
| F34 | kaempferol-3-*O*-(caffeoyl, sinapoyl)triglucoside-7-*O*-glucoside |
| F35 | kaempferol-3-*O*-(feruloyl, methoxycaffeoyl) triglucoside-7-*O*-diglucoside |
| F36 | kaempferol-3-*O*-(*p-*coumaroyl)diglucoside |
| F37 | kaempferol-3-*O*-(disinapoyl) triglucoside-7-*O*-diglucoside |
| F38 | kaempferol-3-*O*-(feruloyl, methoxycaffeoyl)triglucoside-7-*O*-glucoside |
| F39 | kaempferol-3-*O*-(disinapoyl)triglucoside-7-*O*-glucoside |
| F40 | kaempferol-3-*O*-(feruloyl, sinapoyl) triglucoside-7-*O*-diglucoside |
| F41 | kaempferol-3-*O*-(caffeoyl, sinapoyl)triglucoside |
| F42 | kaempferol-3-*O*-glucoside |
| F43 | kaempferol-3-*O*-(feruloyl, sinapoyl)triglucoside-7-*O*-glucoside |
| F45 | kaempferol-3-*O*-(feruloyl, sinapoyl)triglucoside |
| F46 | kaempferol-3-*O*-(diferuloyl)triglucoside |
|  | **Quercetin derivatives** |
| F3 | quercetin-3-*O-* diglucoside -7-*O-*glucoside |
| F4 | quercetin-3,7,4´- tri-*O-*glucoside |
| F9 | quercetin-3-*O-*(caffeoyl, sinapoyl)triglucoside |
| F12 | quercetin-3-*O-*sophoroside-7-*O-*(caffeoyl)glucoside |
| F13 | quercetin-3-*O-*glucoside-7-*O-*(caffeoyl)glucoside |
| F22 | quercetin-3-*O*-glucoside-7-*O*-glucoside |
| F30 | quercetin-3-*O*-(caffeoyl, sinapoyl)triglucoside-7-*O*-glucoside |
